# Harnessing the tunable cavity of nanoceria for enhancing Y-27632-mediated alleviation of ocular hypertension

**DOI:** 10.7150/thno.54525

**Published:** 2021-03-13

**Authors:** Li-Jyuan Luo, Duc Dung Nguyen, Jui-Yang Lai

**Affiliations:** 1 Graduate Institute of Biomedical Engineering, Chang Gung University, Taoyuan 33302, Taiwan.; 2 Department of Ophthalmology, Chang Gung Memorial Hospital, Linkou, Taoyuan 33305, Taiwan.; 3 Department of Materials Engineering, Ming Chi University of Technology, New Taipei City 24301, Taiwan.; 4 Research Center for Chinese Herbal Medicine, College of Human Ecology, Chang Gung University of Science and Technology, Taoyuan 33303, Taiwan.

**Keywords:** ceria nanostructure, shell thickness, hollow carrier system, Y-27632, ocular therapeutics

## Abstract

**Background:** Y-27632 is a potent ophthalmic drug for the treatment of ocular hypertension, a globally prevalent eye disease. However, the sustained delivery of Y-27632 by a therapeutic carrier to lesion sites located in the inner segments of the eye for effectively treating the ocular disorder still remains challenging.

**Methods:** To realize the goal, a strategy based on solvothermal-assisted deposition/infiltration in combination with surface modification is utilized to synthesize hollow mesoporous ceria nanoparticles (HMCNs) with tailorable shell thicknesses and drug release profiles. The shell thickness of HMCNs is rationally exploited for achieving sustained drug release and advanced therapeutic benefits.

**Results:** The shell thickness can regulate release profiles of Y-27632, displaying that thick and thin (~40 nm and ~10 nm) shelled HMCNs reveal burst release characteristics (within 2 days) or limited drug loading content (~10% for the 40 nm thick). As a compromise, the HMCNs with moderate shell thickness (~20 nm) possess the most sustained drug release over a period of 10 days. In a rabbit model of glaucoma, a single instillation of the optimized Y-27632-loaded HMCNs can effectively treat glaucoma for 10 days via simultaneously repairing the defected cornea (recovery of ~93% ATP1A1 mRNA levels), restoring the reduced thickness of outer nuclear layer to normal (~64 µm), and restoring ~86% of the impaired photoreceptor cells.

**Conclusion:** A comprehensive study on the importance of HMCN shell thickness in developing long-acting nano eye drops for the efficient management of glaucoma is proposed. The findings suggest a central role of nanobiomaterial structural engineering in developing the long-life eye drops for pharmacological treatment of intraocular diseases.

## Introduction

With a global prevalence of 3.54% among adults aged between 40 and 80 years, glaucoma has been recognized as the leading cause of blindness in the world 1. High intraocular pressure (IOP) is considered to be the main marker for glaucoma, and lowering the elevated IOP is the first-line of therapeutic strategy to prevent loss of vision in patients with glaucoma 2. Current treatment modalities for the reduction of IOP in glaucoma include laser or incisional surgical as well as drug therapies. It is worth noting that the topical administration of eye drops is considered the most cost-effective treatment, and thus is employed more often, especially in the developing countries. An elevated IOP can cause stress in the corneal stroma, affecting the organization of its collagen fibrils thereby altering the biomechanical properties, structure, and transparency of the cornea 3, 4. Moreover, IOP elevation is signified by an increase in aqueous humor outflow resistance, possibly attributed to alterations in the contractile characteristics of the ciliary muscle and trabecular meshwork (TM) 5, 6. Such alterations are physiologically caused by augmented levels of bioactive molecules in the aqueous humor of glaucomatous eyes, which include reactive oxygen species (ROS), transforming growth factor beta (TGF-β), and interleukins (ILs). These molecules trigger diverse intracellular pathways responsible for cell proliferation, adhesion, and contraction 7-9. Among these pathways, Rho-associated protein kinase (ROCK) signaling plays a pivotal role in the regulation of ciliary muscle and TM cell contraction, cell stiffness, and extracellular matrix arrangement via the phosphorylation of target proteins 10, 11. These changes, in combination with high levels of ROS and inflammatory molecules, can cause apoptosis of the photoreceptor cells and subsequent retinal degeneration, resulting in visual impairment 12-14. Therefore, the development of pharmacological treatments that simultaneously block ROCK signaling pathway activation, inhibit retinal degeneration and promote retinal regeneration, and prevent other accompanying risk factors (e.g. oxidative stress and inflammation) are greatly sought after for the efficient management of glaucoma progression.

Several pharmacological ROCK inhibitors have been developed as therapeutic targets in glaucoma therapy over the past decade 15. One of the most widely investigated ROCK inhibitors is Y-27632, which demonstrates an outstanding ability to reduce IOP. Y-27632 works by modulating cytoskeletal alterations in TM cells, increasing conventional outflow through the TM, and relaxing ciliary muscle contraction 16, 17. As glaucoma is a complex disease that requires longstanding pharmacological treatments, establishing new means for the sustained delivery of Y-27632 in order to reduce further intervention is highly desirable. The delivery of drugs to the eye is commonly carried out using one of three routes of administration: systemic, intraocular, or topical. Systemic delivery is commonly preferred because of its simplicity, however this route is limited by low drug delivery efficiency and often results in adverse effects. Intraocular implantation/injection can provide greater drug delivery efficiencies at lesion sites, but carrying high risks of infection, hemorrhage, and retinal detachment as well as patient discomfort. Topical instillation is therefore considered to be the most noninvasive approach for the treatment of glaucoma, as it causes less discomfort and has increased levels of patient compliance 18. This administration route, however, has certain limitations due to ocular barriers such as tear turnover, nasolacrimal drainage, and reflex blinking, all of which impede the access of drug molecules into the intraocular tissues of the eye 19, 20. An effective strategy in overcoming these obstacles is the utilization of nanoparticles as drug carriers. Their small size and large surface area help to extend drug residence time in the pre-corneal region thereby improving drug bioavailability 21, 22. However, the topical delivery of Y-27632 by nanocarriers to sites of pharmacological action for the effective treatment of glaucoma has not yet been developed.

Among the various types of nanocarriers that have been developed for drug delivery systems, mesoporous nanoparticles have attracted enormous attention owing to their large surface area, controllable porosity, and abundant active sites on the surface 23-25. However, mesoporous nanoparticle-based carrier systems typically have limited drug encapsulation capabilities and exhibit a high initial burst (within a few hours) 26, 27. In order to achieve higher drug loading performance while reducing burst release, there are ongoing efforts to design mesoporous nanocarriers with hollow architectures 28, 29. For example, an increase of ~11% in drug loading efficiency has been reported when using hollow mesoporous silica nanoparticles in comparison to solid counterparts. Of importance is that no obvious burst release of either carvedilol or fenofibrate has been observed in the hollow carriers 26. Furthermore, it has been demonstrated that at a fixed void size (~70 nm), hollow mesoporous silica nanocarriers show different drug release profiles depending on their shell thicknesses, which vary from 20 to 95 nm. A thicker shell is beneficial for the retardation of encapsulated doxorubicin molecules: hollow nanocarriers with a thickness of 95 nm can release 11.7% of doxorubicin, while those with a thinner shell (e.g. 20 nm) release higher levels of 19.7% 28. Shell thickness, therefore, is a key parameter to consider when developing hollow nanocarriers with desirable drug release profiles for specific biomedical applications. More importantly, the therapeutic nature and surface functionality of the carrier materials are of worthy consideration in formulating efficient topical drug carriers 30-32. Inspired by these findings, we seek to develop topical ophthalmic formulations for the improved pharmacological treatment of glaucoma by exploring the effects (at a fixed particle size) of the shell thickness of hollow mesoporous ceria nanoparticles (HMCNs). These possess highly therapeutic characteristics and modified surfaces for targeting intraocular tissues.

In this study, we present a method to explore the effects of HMCN shell thickness on the encapsulation and release of ROCK inhibitor Y-27632 for the treatment of glaucoma. Shell thickness was adjusted through the iterative deposition and infiltration of ceria on the HMCNs, which were synthesized using solvothermal synthesis via a hard template method. The surface of HMCNs with a controlled shell thickness was chemically modified using both chitosan and ZM241385 to facilitate the nanoparticles to penetrate corneal epithelial tight junctions, and to enable the nanoparticles to bind on to the ciliary body and TM tissues. *In vitro* and *in vivo* studies show that surface-modified HMCNs are highly biocompatible with TM cells. Carriers with a greater shell thickness have also been shown to possess a stronger ability to suppress hydrogen peroxide-induced ROS generation and to attenuate the elevated expression of inflammatory molecules (IL-6) in TGF β-stimulated TM cultures. ROCK inhibitor Y-27632, once encapsulated by the surface-modified HMCNs, revealed different release profiles in response to the shell thickness of the HMCNs, demonstrating that the most sustained release performance (over 10 days) could be obtained using carriers with a moderate shell thickness of 20 nm. In an experimental rabbit model of glaucoma, pharmacological treatments relying on a single topical instillation of surface-modified and shell-thickness-controlled HMCNs that encapsulate Y-27632, exhibited a strong correlation between shell-thickness and treatment efficacy. Formulations employing 20 nm-thick shell carriers loaded with Y-27632 were able to effectively mitigate the progression of glaucoma by simultaneously repairing the defective corneal stroma and corneal endothelium and restoring the damaged photoreceptor cells. Our findings, therefore, provide a significant advancement in demonstrating the impact of HMCN shell thickness when developing a long-acting topical ophthalmic formulation for effective glaucoma therapy.

## Methods

### Materials

Cerium(III) nitrate hexahydrate (99% trace metal basis), tetraethylorthosilcate (TEOS), ethanol (anhydrous > 99.5%), ethylene glycol (anhydrous 99.8%), ammonium hydroxide (28% NH_3_ in H_2_O), sodium hydroxide (97%), 1-ethyl-3-(3-dimethyl aminopropyl) carbodiimide hydrochloride (EDC), 4-(-2-[7-amino-2-{2-furyl}{1,2,4}triazolo{2,3-a}{1,3,5}triazin-5-yl-amino]ethyl)phenol (ZM241385), hydrogen peroxide (HP), 2,2'-diphenyl-1-picrylhydrazyl (DPPH), Y-27632, and α-chymotrypsin were purchased from Sigma Aldrich (St. Louis, MO, USA). Phosphonate-PEG-COOH (MW 1200 g mol-1; ref. SP-1P-10-001) was supplied by Specific Polymers (Castries, France). Chitosan (Cat. No. 28191), a commercial powder derived from crab shell, was supplied by Fluka (Milwaukee, WI, USA). All the other chemicals were of reagent grade and used as received.

### Synthesis of surface modified and shell-thickness controlled HMCNs

The HMCNs were prepared using silica nanoparticles as a template and repeated solvothermal reactions as a regulator to control the shell thickness. EDC chemistry was employed to modify the HMCN surface and thereby endow the nanocarriers with targeted delivery function. Detailed information on the synthesis and modification of the functional HMCNs can be found in section 1.1 in the Supplementary Material.

### Material characterization

Transmission electron microscopy (TEM) and X-ray diffraction (XRD) spectroscopy were used for morphological and structural examination of the HMCNs. The surface characteristics were quantified by using zeta potential, specific surface area, ninhydrin assay, and UV-Vis spectroscopy measurements. Detailed information can be found in section 1.2 in the Supplementary Material.

### *In vitro* biocompatibility studies

Human TM cell line was used to assess *in vitro* biocompatibility of the HMCN nanocarriers. Only healthy TM cells from passages 5 through 8 were studied. Phase-contrast microscopy was utilized to observe cellular morphology. Metabolic activity was estimated by a mitochondrial dehydrogenase (MTS) assay. Additionally, genotoxicity in TM cells that exposed to the HMCNs was evaluated using comet assay. Detailed *in vitro* biocompatibility studies can be found in section 1.3 in the Supplementary Material.

### Anti-oxidant and anti-inflammatory activity studies

Anti-oxidant activity of the HMCNs was investigated via measurement of intracellular ROS levels in TM cells at different conditions. Moreover, free radical scavenging activity of HMCNs was also evaluated. Anti-inflammatory activity of the HMCN nanocarriers was determined by enzyme-linked immunosorbent assay (ELISA). Detailed experimental processes can be found in section 1.4 in the Supplementary Material.

### *In vitro* drug release profiles and cellular regulation properties

*In vitro* drug release profiles from different HMCN nanocarriers were obtained using a high-performance liquid chromatography (HPLC). The Y-27632 loading content (%) was calculated as follows: loading content (%) = (W_t_/W_np_) × 100%, where W_t_ is the total weight of Y-27632 entrapped in the HMCNs and W_np_ is the weight of HMCNs (n = 5). Release profiles of Y-27632 from different types of HMCNs were obtained by dispersing 3.0 mg of each type of drug-loaded carriers in 5 mL of a balanced salt solution (BSS) at a controlled pH of 6.5. At predetermined time intervals, release buffer was collected and its Y-27632 amount was analyzed by HPLC (n = 5). The Y-27632-loaded HMCNs were incubated with TM cells to evaluate the shell thickness effects of the nanocarriers on Y-27632 to modulate TM contraction *in vitro*. Western blot analysis was used to assess the shell thickness effects on the dephosphorylation of myosin light chain 2 (MLC2) and restoration of cofilin activity. Detailed experiments on the *in vitro* release and cellular regulation can be found in section 1.5 in the Supplementary Material.

### Shell thickness effects on glaucoma therapy

Adult New Zealand white rabbits (National Laboratory Animal Breeding and Research Center, Taipei, Taiwan, ROC) with weight in the range of 3.0-3.5 kg and age between 16 and 20 weeks were employed for pharmacological treatment studies. All animal procedures were approved by the Institutional Animal Care and Use Committee and were conducted following the ARVO Statement for the Use of Animals in Ophthalmic and Vision Research. The animals were first anesthetized by intramuscular injections of tiletamine hydrochloride/zolazepam hydrochloride mixture (2.5 mg/kg body weight, Zoletil; Virbac, Carros, France) and xylazine hydrochloride (1 mg/kg body weight, Rompun; Bayer, Leverkusen, Germany). A total of 24 glaucomatous rabbits were successfully induced by α-chymotrypsin (0.1 mg/mL) injection to the posterior chamber of the eye. All the as-induced glaucomatous rabbits were classified into glaucomatous (GL) group, while six glaucomatous rabbits were left untreated during the follow-up period and categorized as progressively glaucomatous (Ctrl) group. For the three test groups (CNP10nm, CNP20nm, and CNP40nm) of rabbits (6 rabbits/group), the glaucomatous animals received topical instillation of 50 μL of each Y-27632-loaded HMCN solution, which was prepared by mixing 2% w/v Y-27632 with 1 mg/mL of HMCNs. At preoperation (Pre), all the animals were normal and free of ocular surface disease as clinically diagnosed. A follow-up period of 10 days was executed. IOP and electroretinogram (ERG) measurements were performed on the test animals after being anesthetized. Y-27632 and ceria resided in the cornea, ciliary body, and retinal tissue of test rabbit eyes were characterized using inductively coupled plasma mass spectrometry and HPLC methods. Morphology, structure and mechanical properties of corneal tissues were analyzed using scanning electron microscopy (SEM), TEM, and Young modulus measurement. Hematoxylin and eosin (H&E) staining and terminal deoxynucleotidyl transferase (TdT)-mediated dUTP nick end labeling (TUNEL) assay were applied on retinal tissues for assessment of shell thickness effects on the retinal degeneration caused by glaucoma. Detailed experiments can be found in section 1.6 in the Supplementary Material.

### Statistical analysis

Data were expressed as mean ± standard deviation (SD). Comparative studies of means were performed using a one-way analysis of variance (ANOVA) followed by a Newman-Keuls post hoc test. Significance was accepted with P < 0.05.

## Results and Discussion

Various synthetic approaches have been proposed for the preparation of ceria nanoparticles for biomedical application, due to the promising therapeutic effects gained from their anti-oxidant 33, anti-inflammatory 34, and neuroprotective 35 properties. Suggested techniques include flame spraying, thermal decomposition, and solvothermal precipitation 36. Our method for fabricating HMCNs with adjustable shell thicknesses is based on a modified solvothermal precipitation technique that includes two main steps: i) solvothermal precipitation of the ceria onto silica nanospheres, followed by removal of the templates to achieve pre-fabricated HMCNs; ii) iterative deposition and infiltration of the ceria into the interior spaces of the pre-fabricated HMCNs, under the same solvothermal conditions.

**Figure 1:**
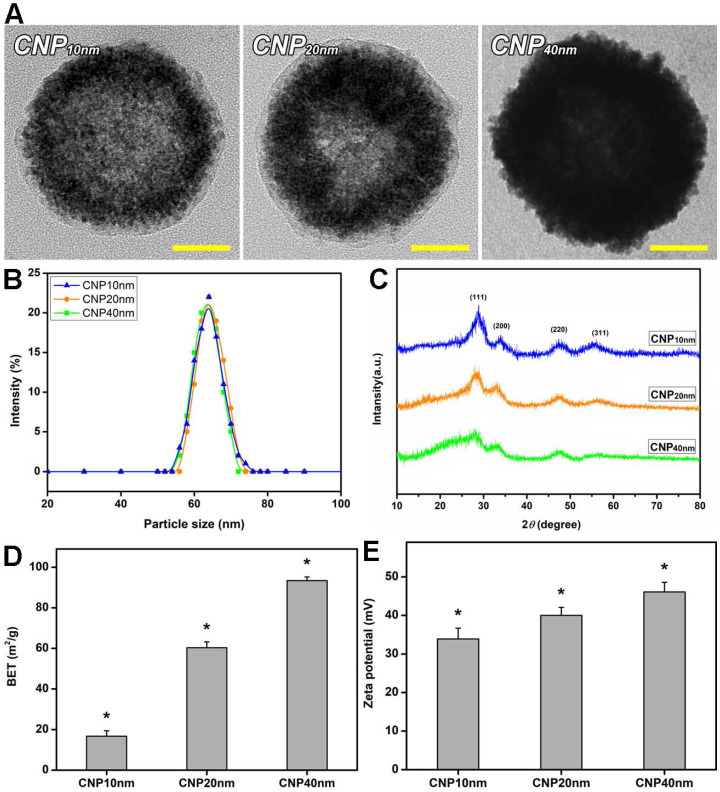
** Material characterization.** (A) Representative TEM images, (B) DLS measurements, (C) XRD patterns, (D) BET surface areas, and (E) zeta potentials of surface-modified HMCNs fabricated using different solvothermal deposition and infiltration times. Values are mean ± SD (n = 5). ^*^P < 0.05 vs all groups. Scale bars: 20 nm.

To endow the shell-thickness-controlled HMCNs with targeted delivery functions, chemical modifications were carried out on their surfaces. These modifications included the attachment of phosphonate polyethylene glycol (PEG), containing COOH terminal functionality, and its functionalization with chitosan and ZM241385 using EDC coupling chemistry 37. Figure 1A shows typical TEM images of the functional HMCNs, fabricated using different solvothermal deposition and infiltration times. All the HMCNs had comparable lateral particle sizes but different shell thicknesses. The polymeric coating (chitosan and ZM241385) is also revealed on the particle surfaces. The shell thicknesses for HMCNs fabricated by exposing them to 1×, 3× and 5× solvothermal deposition and infiltration were ~10, 20, and 40 nm, respectively. The increase in shell thickness caused by increases in solvothermal iteration can possibly be explained by the preferable diffusion of cerium nitrate into hollow voids through the porous channels of the pre-formed shells of pre-fabricated HMCNs. Under solvothermal conditions, the inward-diffused and confined cerium nitrate can be quickly precipitated as additional ceria on the inner shells, increasing their thickness, possibly due to its high physical affinity to the pre-formed shell surfaces. In contrast, the cerium nitrate molecules outside of the hollow voids are highly free to move through the entire solvothermal solution and reactors. The probability of their precipitation on the exterior shells is, therefore, much lower and may be negligible in comparison to the inward-diffused molecules.

The surface-bound amine group for HMCNs with a shell thickness of 10 nm (CNP10 nm), 20 nm (CNP20 nm), and 40 nm (CNP40 nm) were 207.6 ± 9.3, 225.7 ± 7.4, and 249.8 ± 9.8 μmoL/mg NP respectively, implying a slight increase in the amount of chitosan produced as shell thickness increased (Table S1). A similar trend was also observed in the ZM241385 grafting amount: the values for CNP10 nm, CNP20 nm, and CNP40 nm were 6.9 ± 1.2, 11.2 ± 1.5, and 16.4 ± 0.9 μg/mg NP respectively (Table S1). The increase in the amount of surface modified materials as shell thickness increased can be attributed to the greater active sites (more ceria materials) of the thicker hollow particles. Results from dynamic laser scattering (DLS) measurement of the surface of modified and shell-thickness controlled HMCNs are shown in Figure 1B and Table S1. No obvious difference in particle-size distribution was found, showing good agreement with TEM data. Figure 1C indicates the XRD patterns of different types of HMCNs, fabricated using various solvothermal iteration times. All patterns indicate characteristic diffraction peaks assigned to the (111), (200), (220), and (311) lattice planes of a typical fluorite cubic CeO2 structure (JCPDS No. 34-0394). As the iteration increased, broader characteristic peaks were observed, suggesting the enrichment of polycrystalline degrees, possibly attributed to the interruption of continuous ceria deposition during the iterative fabrication process. The Brunauer Emmett Teller (BET) measurement was also used to evaluate the specific surface areas of the HMCNs (Figure 1D). As anticipated, the highest BET surface area was obtained for the CNP40 nm group whereas the CNP10 nm group possessed the lowest BET value: the specific surface areas were 16.7 ± 2.7 m2/g, 60.3 ± 2.9 m^2^/g, and 93.4 ± 1.8 m^2^/g for CNP10nm, CNP20nm, and CNP40 nm, respectively. The zeta potentials of the HMCNs (Figure 1E) were measured to be 33.9 ± 2.8 mV (CNP10 nm), 40.0 ± 2.1 mV (CNP20 nm), and 46.1 ± 2.5 mV (CNP40 nm), revealing the positive charge nature and high stability of all of the particle dispersions. Prior to evaluating the effects of HMCN shell thickness on their potential use as ophthalmic drug delivery systems, it is necessary to examine their ocular biocompatibility. Therefore, the effects of shell thickness on the *in vitro* biocompatibility of HMCNs with TM cells were investigated using phase-contrast microscopy, mitochondrial dehydrogenase (MTS) assays, and alkaline comet assays. Figure S1A shows phase-contrast images of TM cells after exposure to various types of HMCNs for two days. For comparison, cell cultures incubated without any types of HMCNs were used as the control group. All of the cells incubated with different types of HMCNs exhibited a typical behavior and healthy morphology, analogous to those in the control group, suggesting that all of the examined HMCNs were highly biocompatible with ocular cells. To support these qualitative phase imaging results, the MTS activity assay was employed to evaluate the metabolic activity of TM cells. No obvious changes in the MTS activity (~93-100%) of the cell cultures with or without HMCN treatments were found (Figure S1B), quantitatively confirming the outstanding biocompatibility of the HMCNs to the ocular cells. In addition, the alkaline comet assay was utilized to assess the possible genotoxicity of the HMCNs. Fluorescence microscopy images (Figure S1C) indicate intact nuclei with smooth margins in all test groups. The quantification of DNA damage in TM cells incubated with different types of HMCNs (Figure S1D) suggests that shell thickness exerts a negligible genotoxicity, as signified by comet tail lengths comparable to those in the control group.

**Figure 2:**
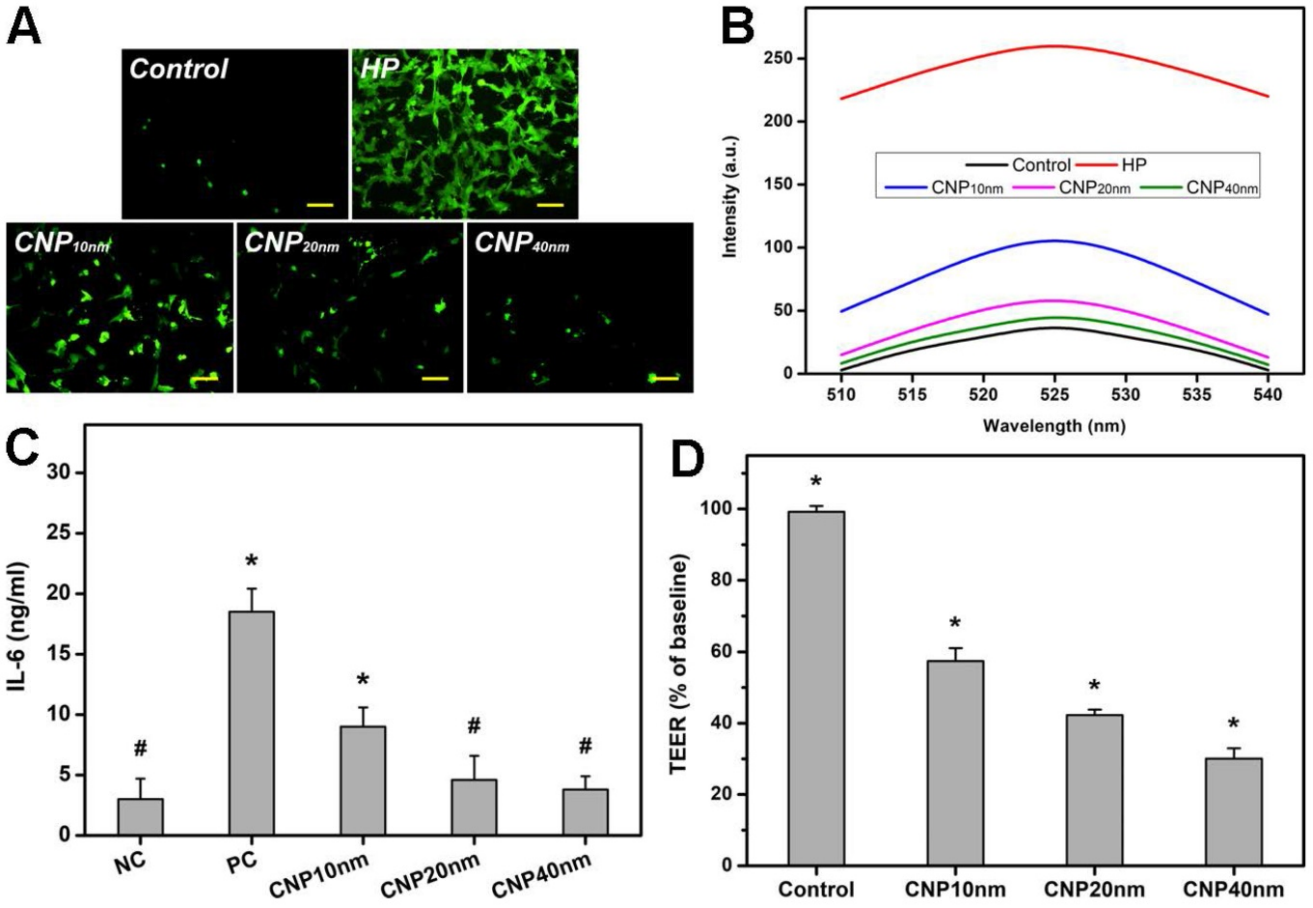
***In vitro* cellular studies.** (A) Effect of different types of HMCNs on suppression of H_2_O_2_-induced intracellular ROS production in TM cells. TM cells were incubated with test HMCNs for 24 h and further exposure to H_2_O_2_ for 24 h; cells incubated with no materials served as Control. Scale bars: 100 µm. (B) Intracellular levels of ROS were measured by the fluorescence intensity of DCFH-DA, with a microplate reader. (C) Levels of IL-6 released from TM cultures after incubation with test samples for 3 days. Unstimulated and TGF-β-stimulated cells without contacting the test HMCNs were the negative control (NC) and positive control (PC). Values are mean ± SD (n = 6). ^*^P < 0.05 vs NC, CNP20 nm and CNP40 nm groups; ^#^P <0.05 vs PC and CNP10 nm groups. (D) TEER values of rabbit corneal endothelial cells exposed to different types of HMCNs for 24 h. Control: without materials. Values are mean ± SD (n = 6). ^*^P < 0.05 vs all groups.

As oxidative stress and inflammation play central roles in the pathogenesis of glaucoma, and elevated levels of ROS and interleukins can induce retinal degeneration 38, 39, the utilization of nanocarriers with desirable anti-oxidant and anti-inflammatory properties is highly expected to address the progression of the disease. In this regard, the effects of HMCN shell thickness on their anti-oxidant and anti-inflammatory capabilities were investigated *in vitro*. Figure 2A depicts representative fluorescence images of ROS production in TM cultures incubated with different types of HMCNs, incorporated with hydrogen peroxide (test groups), hydrogen peroxide only (HP group), and no hydrogen peroxide (control group). A minimal level of green fluorescence signals can be observed in the control group while the strongest fluorescence intensity is shown in the HP group, suggesting that hydrogen peroxide can induce a high expression of ROS in the cell cultures. As the shell thickness increased from 10 to 40 nm, a gradual decrease in the fluorescence signal was perceived. This result implies that HMCNs with thicker shells have stronger capabilities in attenuating ROS production induced by hydrogen peroxide, probably due to the contribution of their greater amounts of ceria 40. Quantitative levels of ROS production were further analyzed by spectrofluorometer (Figure 2B). Similar fluorescence profiles were attained in all groups, but their intensities were remarkably different. The ascending ranking of intracellular ROS production was as follows: Control < CNP40 nm < CNP20 nm < CNP10 nm < HP, demonstrating the clear effects of HMCN shell thickness on ROS inhibition. The shell thickness-dependent antioxidant property was further reinforced by the free radical scavenging activities of the tested HMCNs (Table S1), confirming that the thicker shelled particles provide stronger antioxidant properties.

To investigate whether shell thickness could influence the anti-inflammatory activity of the HMCNs, TGF-β-stimulated TM cells were incubated with different types of HMCN solutions. The production of IL-6 was analyzed (Figure 2C), as this cytokine plays a key role in mediating the inflammation associated with glaucoma 41. The results demonstrated that the IL-6 level was low (3.0 ± 1.7 ng/mL) in healthy cell cultures (the NC group) and considerably higher (18.5 ± 1.9 ng/mL) in TGF-β-stimulated TM cells (the PC group), implying the successful induction of inflammation. The IL-6 levels in the inflammatory cells treated with various types of HMCNs differed in response to the shell thickness of the HMCNs. All of the test groups exhibited reduced IL-6 levels in comparison to the PC group and the cytokine levels of the CNP20nm and CNP40 nm groups in particular were as low as those of the NC group. Similar results for the suppression of IL-6 production have been previously reported, demonstrating that ceria nanoparticles can downregulate the production of IL-6 in mouse myocardium in a murine model of cardiomyopathy 42. In accordance with this finding, our data further suggest the impact of shell thickness on the achievement of effective attenuation of elevated IL-6 levels in inflammatory cells. The transepithelial electrical resistance (TEER) integrity of tight junction dynamics in a cell culture model of an epithelial monolayer was also measured. The TEER values (an indicator for epithelial tightness) for the untreated TM cell layer (control group) were close to the baseline level. In the HMCN-treated groups, a gradual decrease in TEER values was obtained as the shell thickness increased (Figure 2D). These results reflect the clear effects of shell thickness on the opening of epithelial tight junctions, demonstrating that HMCNs with a thicker shell possess a stronger ability to open tight junctions, signified by lower TEER values (i.e., more ions passing through the paracellular route).

**Figure 3:**
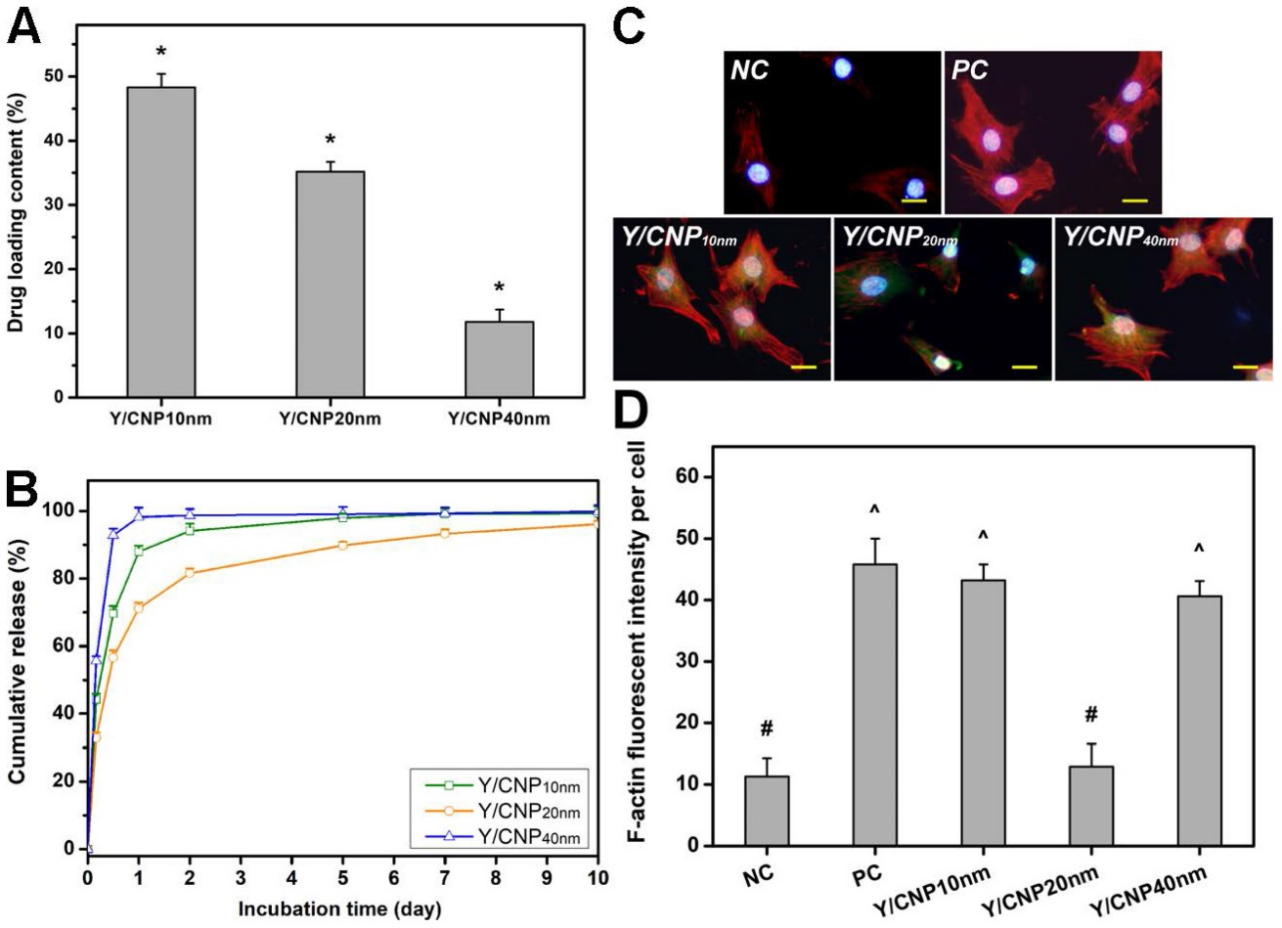
***In vitro* drug release and cellular regulation studies.** (A) Y-27632 drug loading content. Data are mean ± SD (n = 5); ^*^P < 0.05 vs all groups. (B) Cumulative drug release profiles from various types of HMCNs. (C) Immunofluorescence staining with DAPI (blue fluorescence), F-actin (red fluorescence) and FITC-HMCNs (green fluorescence) and (D) mean fluorescence intensity of the TM cells after incubation with test materials for 5 days. Scale bars: 10 µm. Values are mean ± SD (n = 6). ^#^P < 0.05 vs PC, Y/CNP10 nm and Y/CNP40 nm groups; ^^^P < 0.05 vs NC and Y/CNP20 nm groups.

The effects of HMCN shell thickness on their drug delivery performance was investigated *in vitro* using Y-27632 as a model drug. The drug molecules were diffused into the hollow spaces of the HMCNs through porous channels on their shells, and the drug entrapment inside the HMCNs was controlled by pH-stimulation 43. Figure 3A shows the drug loading content of HMCNs with different shell thicknesses. The loading capacities of the HMCNs were 48.0 ± 1.7% (Y/CNP10 nm), 34.3 ± 2.8% (Y/CNP20 nm), and 9.6 ± 2.0% (Y/CNP40 nm), respectively. The obtained data imply a strong correlation between shell thickness and drug loading content, demonstrating that HMCNs with thinner shells can carry a larger amount of the drug. This trend of decreasing drug loading content as the shell thickness of the HMCNs increases can be attributed to shrinking of the hollow spaces (i.e., reduction of reservoir sizes) as well as to the elevation of positive charges (Figure 1), which may impede the access of positively charged Y-27632 molecules 44.

The Y-27632-loaded HMCNs were then examined in BSS solutions with a pH of 6.5, as this value is believed to be low enough to allow the polymeric coating to be swollen for the release of drug molecules. BSS solutions with much lower pH values are inappropriate for mimicking the aqueous humor of glaucomatous eyes (pH of less than 6.65) 45. Figure 3B shows the cumulative drug release profiles of different HMCN carriers, demonstrating different release characteristics in response to shell thickness. Most of the drug molecules encapsulated by the thickest shell HMCNs were released after one day (the Y/CNP40 nm group). In the CNP10nm group, the amount of drug released was 88% on day one and 93% on day two, signifying an improved drug release performance in comparison to the thickest shell carriers. Remarkably, the HMCN carriers with moderate shell thicknesses (the CNP20 nm group) exhibited the most sustained drug release profile over a long follow-up period of up to ten days. The release percentages for this group were 71% on day one, 81% on day two, and 96% on day ten. This discrepancy in drug release performances can be ascribed to the shell thickness of the HMCNs as well as the amount of chitosan coating. The HMCNs with the thickest shells (CNP40 nm) have limited hollow space for drug storage and thereby the drug molecules are likely located only on the surface of the carriers, consequently being released quickly (burst release). Although the HMCNs with the thinnest shells (CNP10 nm) have the highest drug loading capacity, their ultrathin sheaths are highly favorable for the outward diffusion of drug molecules at faster rates (i.e., with shorter diffusion paths). Moreover, under the mildly acidic condition, the amino groups of chitosan on the HMCNs are protonated 43, resulting in the formation of positively charged surfaces of the chitosan coating. These positive charges on the backbone chains can induce the swelling of the chitosan coating via electrostatic repulsion. As a result, the Y-27632 drug molecules entrapped in the hollow spaces of the HMCNs can diffuse out through the chitosan coating, and therefore the amount of chitosan coating is considered an important factor in regulating the drug release. It is noting that CNP10 nm possessed lower chitosan amount (thinner chitosan coating) as compared to that of the CNP20 nm (Table S1), accordingly drug molecules in the HMCNs with 10 nm shell thickness could be released faster (due to the thinner barrier.) For the CNP40 nm, the limited hollow spaces, thus most drug molecules absorbed on the carrier surfaces at minimal amount, are the main factor that induce the burst release. With a trade-off between drug loading capacities, diffusion paths, and chitosan coating amounts, the 20nm thick shelled HMCNs are considered the most beneficial carriers in providing sustained drug release performance. These results reflect the important role of shell thickness when developing efficient drug carriers for glaucoma pharmacotherapy.

Given that Y-27632 drug molecules can induce the reduction of actin secreted by TM cells (i.e., reducing TM tissue contraction and thereby increasing aqueous outflow in eyes with an elevated IOP) 46, it is important to understand the effects of the shell thickness of HMCNs loaded with Y-27632 on the alteration of TM cells. Figure 3C shows fluorescence microscopy images of a healthy TM cell culture (NC group) and TGF-β-stimulated TM cell cultures incubated with different types of HMCNs (test groups) and with no material (PC group). The NC group produced the weakest F-actin (red) fluorescence signal while the strongest F-actin fluorescence signal was found in the PC group, demonstrating that cytoskeletal alteration was successfully induced by TGF-β 47. There were also differences in the F-actin fluorescence signal of the TGF-β-stimulated TM cell cultures after 24 h of treatment with various types of Y-27632-loaded HMCNs. A slight reduction in intensity of the F-actin fluorescence signal in the Y/CNP10nm group, in comparison to the PC groups, suggests a negligible impact from the HMCNs with the thinnest shells (10 nm), possibly due to their burst release of Y-27632 and a low therapeutic amount of ceria. The further decline of the F-actin florescence signal in the Y/CNP40nm groups reflects their superior drug release performance and higher therapeutic amount of ceria gained from a thicker shell. Significantly, the Y/CNP20 nm group exhibited a remarkable reduction in the F-actin signal down to a level close to that of the NC group. This observation demonstrates the high effectiveness of the moderately thick shelled HMCNs, which provide a sustained release of Y-27632 and therapeutic activity in the stimulated cell cultures, consequently preventing the alteration of cytoskeletons in the TM cells. This qualitative estimation of the effects of the shell thickness of HMCNs on their alteration of TM cells is further supported by quantitative analysis using fluorescence intensity (Figure 3D). Collectively, our results demonstrate the important role of HMCN shell thickness in reducing F-actin in TM cells, opening up possibilities for their uses in increasing aqueous humor outflow in glaucomatous eyes.

**Figure 4:**
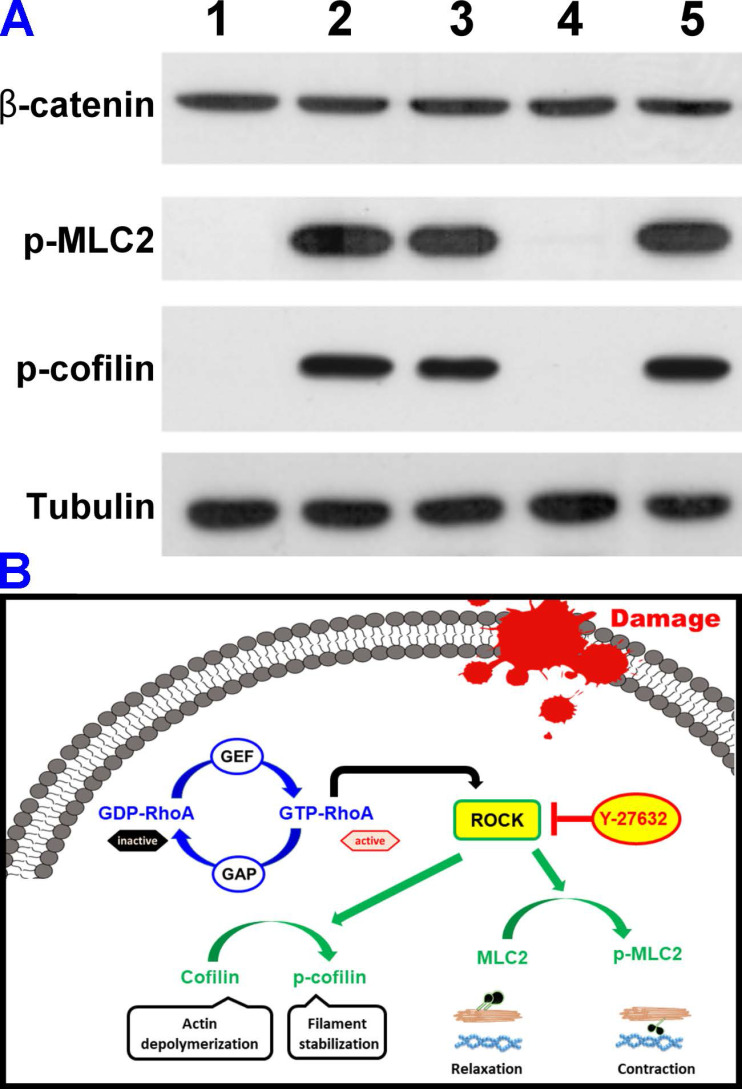
***In vitro* therapeutic studies.** (A) Western blot analysis of β-catenin, phosphorylated MLC2 (p-MLC2), phosphorylated cofilin (p-cofilin), and tubulin in healthy TM cells (lane 1, NC group) and TGF-β-stimulated TM cells untreated (lane 2, PC group) and treated (lane 3-5, test groups) with different types of Y-27632-loaded HMCNs. (B) Schematic model of TGF-β-stimulated ROCK signaling pathway and inhibition in TM cells by the Y-27632-loaded HMCNs with shell thickness of 20 nm.

It has been demonstrated that the disassembly of actin stress fibers and reduction of cell contraction can be achieved through the dephosphorylation of myosin light chain 2 (MLC2), which inhibits ROCK pathways thereby increasing aqueous humor outflow (i.e., reducing elevated IOP) in glaucomatous eyes 16, 48, 49. Furthermore, ROCK is also reported to involve the inactivation of the actin-depolymerizing factor cofilin in TM cells 50. Accordingly, understanding the effects of the shell thickness of HMCNs loaded with Y-27632 on the dephosphorylation of MLC-2 and the restoration of cofilin activity, which depolymerizes actin in TGF-β-stimulated TM cells, is of significant importance in the development of efficient drug carriers for glaucoma therapy. Considering that TGF-β is a key modulator in altering the actin cytoskeleton of TM cells 51, we employed this growth factor to stimulate actin changes in TM cells and to investigate their response to Y-27632-loaded HMCNs with different shell thicknesses. Figure 4A shows a western blot analysis of β-catenin, phosphorylated MLC2 (p-MLC2), phosphorylated cofilin (p-cofilin), and tubulin in healthy TM cells (lane 1, NC group) and TGF-β-stimulated TM cells without (lane 2, PC group) and with (lane 3-5, test groups) treatments using different types of Y-27632-loaded HMCNs. The results indicate that the presence of p-MLC2 and p-cofilin was not detected in healthy TM cells, whereas strong signals were obtained for these two proteins in the stimulated cells, confirming the successful activation of ROCK signaling by TGF-β. Different expressions of the phosphorylated proteins were achieved for stimulated TM cells incubated with various types of HMCNs loaded with Y-27632, further demonstrating the impact of particle shell thickness. In comparison to the PC group, reduced expressions of p-MLC2 and p-cofilin were observed in the Y/CNP10nm and Y/CNP40 nm groups. Significantly, a negligible amount of the two proteins could be detected in the Y/CNP20 nm group. To support this observation, the relative β-catenin, p-MLC2, and p-cofilin levels were quantified (Figure S2). The results further confirm that the HMCNs with a shell-thickness of 20 nm perform best in dephosphorylating MLC-2 and p-cofilin through the depolymerization of actin in TGF-β-stimulated TM cells. This is possibly due to their increased ability to sustain the release of Y-27632 and thereby effectively inhibit ROCK pathways 51. Based on these results, we built a schematic model showing the TGF-β-stimulated ROCK signaling pathways and the inhibition in TM cells by the Y-27632-loaded HMCNs (Figure 4B). Specifically, GTP-RhoA (ROCK)-mediated signaling events were associated with the regulation of MLC phosphorylation and actin cytoskeletal arrangement. The TGF-β triggered GTP-RhoA activated ROCK, which then phosphorylated MLC2 and cofilin. Treatment using Y-27632-loaded HMCNs with shell-thicknesses of 20 nm can therefore promote the sustained inhibitory activity of Y-27632, resulting in dephosphorylation of the phosphorylated MLC2/cofilin. Elevated and reduced degrees of MLC2 and cofilin phosphorylation induce contraction and relaxation responses, accordingly affecting actin cytoskeletal arrangement and cell morphology, which is believed to be the key determinant in regulating high IOP in glaucomatous eyes.

To evaluate the effects of HMCN shell thickness on their use as drug carriers for glaucoma therapy, an experimental rabbit model of glaucoma was examined *in vivo*, induced by α-chymotrypsin and eye drop formulations containing different types of HMCNs loaded with Y-27632. For clinical observation, the IOP of the glaucomatous eyes receiving a topical instillation of different Y-27632-loaded HMCN dispersions was measured (test groups). Glaucomatous eyes receiving no treatment served as the Ctrl group for comparison. Preoperation (Pre), the IOP values in normal rabbits were near the baseline, whereas rabbits induced with glaucoma (GL) had elevated IOP values of 22.5 ± 1.8 mmHg (Figure S3). The IOPs of the Ctrl group were maintained at high levels during follow-up observations and gradually increased to 27.5 ± 1.6 mmHg on day 10.

Treatment using various formulations of Y-27632-loaded HMCNs also revealed different IOP profiles in response to different shell thicknesses. The formulation using HMCNs with a thickness of 40 nm (the Y/CNP40 nm group) was moderately effective, resulting in slightly lower IOP values in comparison to those of progressively glaucomatous eyes (control group). The low IOP values (near the baseline) of the Y/CNP10nm group suggest that improved treatment efficacy could be achieved. However, the effective period was only for 5 days, possibly due to the moderately limited release profile of HMCN carriers with a cell thickness of 10 nm. Significantly, the Y-27632-loaded HMCN carriers with a shell thickness of 20 nm could effectively lower the high IOP values of the diseased eyes to normal values after four hours and maintain them close to the baseline for ten days. A similar trend in regulation of IOP profiles by the Y-27632-loaded HMCNs was also observed in the glaucomatous eyes induced using latex microspheres (Figure S4), confirming the reproducible efficacy of the formulations. This effective pharmacological treatment can be attributed to the sustained drug release and targeted delivery of the therapeutic HMCNs to the pharmacological sites of action (TM and ciliary body tissues). It is worth noting that topical administration of the Y-27632 drug or HMCN carriers alone could only reduce the elevated IOP to 12.2 ± 1.4 mmHg or 20.1 ± 1.2 mmHg at 4 h post-instillation, respectively. The short effective time of the Y-27632 drug can be attributed to its low bioavailability caused by clearance action of the eye, while the poor treatment efficacy of the HMCN carriers only is possibly due to their non-anti-hypertensive nature. These results suggest the synergistic effects between the HMCN carrier and Y-27632 drug could be achieved, thereby the drug-loaded HMCNs were selected as the test formulations for subsequent *in vivo* experiments to correlate the carrier shell thickness with treatment efficacies. To support the hypothesis that the carrier can penetrate through the cornea and reach the ciliary body and TM tissues, mass concentrations of Ce and Y-27632 in the cornea and ciliary bodies of treated rabbit eyes were analyzed at ten days postoperatively (Table 1). As expected, the HMCN carriers with a moderate shell thickness (the Y/CNP20 nm group) revealed the highest mass concentrations of both Ce and Y-27632 at the pharmacological sites of actions, probably attributed to the balance between chitosan and ZM241385 coatings (Table S1) as well as to the Y/CNP20nm group having the best drug delivery performance (Figure 3). In addition, time course of ceria levels in the ocular tissues were also recorded (Figure S5-S7). The peak concentrations in the cornea were obtained at 4 h while those in the ciliary body and retina tissues were reached at 2 d post-administration for all test HMCN formulations. The highest ceria concentration of Y/CNP40 nm in the cornea at the early time points can be ascribed to the largest positive surface charge of the 40 nm-thick-shelled HMCN carriers (Figure 1E), which are preferably bonded to negatively charged surface of the cornea to the greatest extent via electrostatic interaction. Similar trends in the expression of ceria concentrations were also observed in the ciliary body, confirming the key role of the targeting functional material that helps to increase the accumulation of ceria in the ciliary body tissues with increasing ZM grafting amounts (Table S1). In contrast, in the retina, ceria concentration is highest for the Y/CNP10 nm, possibly due to the small size of the 10 nm-thick-shelled HMCN carriers and their minimal amount localized in the cornea and ciliary body tissues. These results further suggest the trade-off effects of the 20 nm-thick shelled HMCNs (CNP20 nm) in achieving effective delivery of both ceria and Y-27632 for efficient management of glaucoma.

An ERG measurement was carried out to assess the effects of HMCN carrier shell thickness on the treatment of glaucomatous eyes. Figure S8A shows the ERG spectra of healthy (Pre group), glaucomatous (GL group), progressively glaucomatous (Ctrl group) and treated glaucomatous (test group) eyes after ten days of single topical administration of different Y-27632-loaded HMCN formulations. There are negligible differences in the waveform profiles of each group, but clearly different amplitudes. Quantitative results (Figure S8B) from the ERG spectra show that a-wave and b-wave amplitudes of the healthy (Pre) eyes are 63.8 ± 2.9 and 266.1 ± 2.7 µV, respectively. In the Ctrl groups, the a-wave and b-wave values decreased to 50.1 ± 2.1 and 229.3 ± 3.8 µV, respectively, suggesting a considerable loss of the electrophysiological function of the retina as well as photoreceptor degeneration 52, 53. Topical instillation using HMCNs with a shell thickness of 10 nm and 40 nm respectively did not increase the amplitude values to greater levels, in comparison to those of the Ctrl groups, indicating the negligible treatment effectiveness. In contrast, pharmacological treatment employing the Y-27632-loaded HMCNs with a shell thickness of 20 nm resulted in significant increases in the amplitudes, reaching the levels of the healthy eyes (Pre group). These data suggest that ERG response is highly correlated to the shell thickness of the HMCNs, demonstrating that those with a moderate thickness of 20 nm are the most effective carriers for delivery of Y-27632 to the intraocular tissues (TM, ciliary body, and retina), for efficient management of glaucoma.

**Figure 5:**
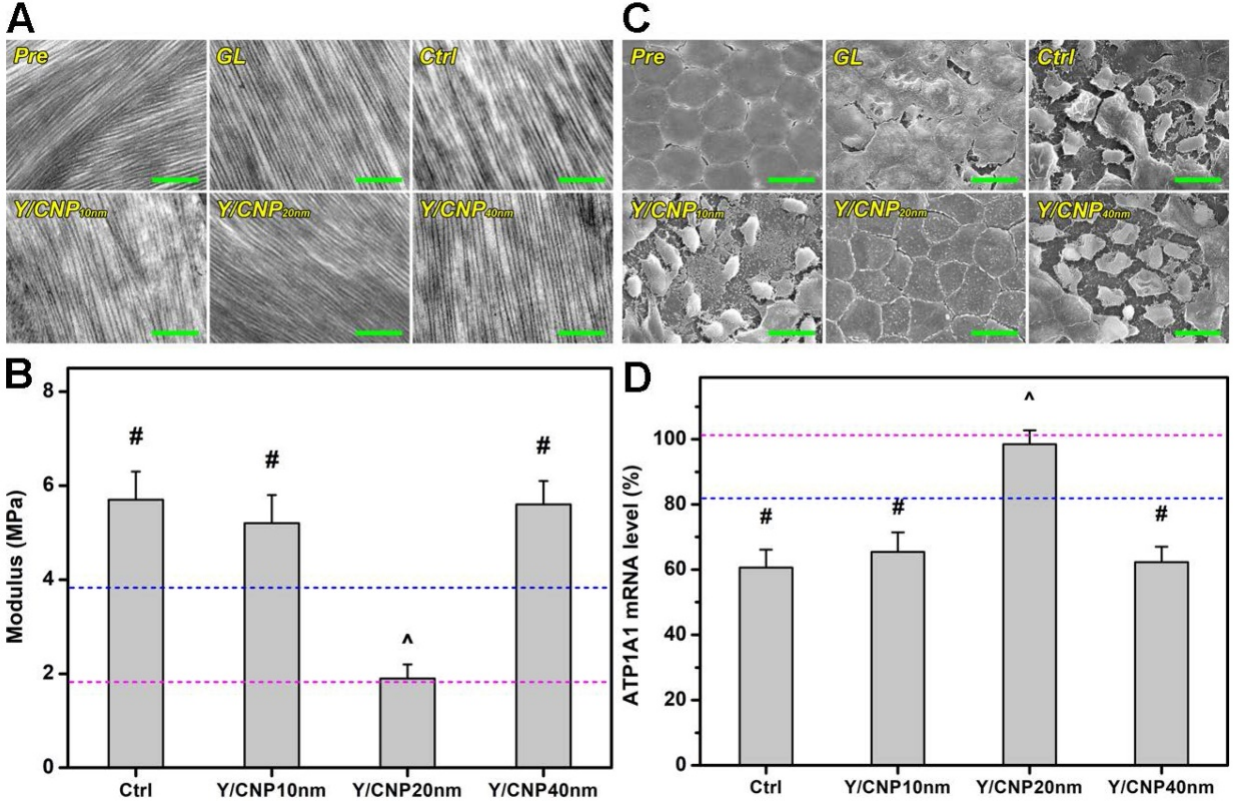
***In vivo* studies on shell thickness effects on corneal structure of glaucomatous eyes.** (A) Typical TEM images of corneal stroma from different rabbit eyes. Scale bars: 1 µm. (B) Young's modulus of corneal tissues; pink and blue dotted lines showing the values of Pre and GL groups, respectively. Values are mean ± SD (n = 6). ^#^P < 0.05 vs Pre, GL and Y/CNP20 nm groups; ^^^P < 0.05 vs GL, Ctrl, Y/CNP10 nm and Y/CNP40 nm groups. (C) Representative SEM images of endothelium of different rabbit eyes. Scale bars: 20 µm. (D) ATP1A1 mRNA levels from endothelium tissues of different rabbit eyes; pink and blue dotted lines showing the values of Pre and GL groups, respectively. Values are mean ± SD (n = 6). ^#^P < 0.05 vs Pre, GL, and Y/CNP20 nm groups; ^^^P < 0.05 vs GL, Ctrl, Y/CNP10 nm and Y/CNP40 nm groups. Ocular tissues were harvested from test rabbit eyes at 10 days post single administration (50 µL of Y-27632-loaded HMCN solution, a mixture of 2% w/v Y-27632 and 1 mg/mL of HMCNs).

As the corneal stroma plays an important role in protecting the inner components of the eye, an elevated IOP could stress the corneal stroma and thereby induce changes in the biomechanical properties of the cornea 3. Research has shown that the normal corneal stroma consists of ordered, parallel collagen fibrils, which maintain corneal shape and transparency 4. Therefore, transmission electron microscopy (TEM) was employed to investigate the effects of the shell thickness of Y-27632-loaded HMCNs on the morphology and arrangement of collagen fibrils in the corneal stroma of glaucomatous eyes receiving the topical administration of different drug-loaded HMCN formulations. Figure 5A shows TEM images of the corneal stroma of different rabbit eyes. It reveals that the corneal stroma in Pre group was comprised of densely and regularly packed collagen fibrils, as generally observed in the normal stroma 54. In the stromal tissues of glaucomatous eyes (GL group), the collagen fibrils became straighter with enlarged interspaces between the fibril bundles, possibly due to increased stress induced by the elevated IOP of the diseased eyes. As the disease progressed (Ctrl group), the collagen fibrils were partially damaged and shortened, and the degree of fibril straightening and interspace sizes were further increased, distorting the collagen fibrils more severely. Topical administration of different Y-27632-loaded HMCN formulations demonstrated the considerable impact of shell thickness on regulating the rearrangement of collagen fibrils in the corneal stroma of glaucomatous eyes. The formulation using the 10 nm thick HMCN carriers could not repair the defective collagen fibrils, whereas the formulation utilizing the thickest shell counterparts (Y/CNP40 nm group) was able to restore and reduce both the lengths and interspace sizes of the fibrils. Interestingly, the restoration of the collagen stromal tissues, in terms of fibril density, morphology, length, and interspaces, was also achieved for the Y/CNP20 nm group.

Young's modulus of the corneal tissues of rabbit eyes was also measured. Figure 5B indicates that the values of Young's moduli for the healthy (pink dashed line), glaucomatous (blue dashed line), and progressively glaucomatous corneas were 1.8 ± 0.6, 3.8 ± 0.4, and 5.7 ± 0.7 MPa, respectively, suggesting that an elevated IOP can cause increases in mechanical strength. It has also been reported that a high IOP in rat eyes can induce a greater mechanical strength in the cornea. This can be ascribed to collagen fibrils in the cornea, which undergo strain-induced stiffening as a result of the high IOP, and produce a higher modulus compared to that of the normal cornea 55. The topical treatments of glaucomatous corneas that used Y-27632-loaded HMCN formulations exhibited different results. The Young's moduli for the diseased corneas in the Y/CNP10 nm and Y/CNP40 nm groups were 5.2 ± 0.6 and 5.6 ± 0.5 MPa, respectively, indicating negligible impacts on the glaucomatous eyes. The pharmacotherapy based on HMCNs with a shell thickness of 20 nm lead to a Young's modulus close to that of healthy tissue (Pre group), showing the effective attenuation of a high IOP, preventing the progression of glaucoma as a consequence. In addition to IOP reduction capability, Y-27632 has been reported to be capable of inhibiting the transition of keratocytes into myofibroblasts and causing the cells in the damaged rabbit cornea to take on a partial embryonic characters, consequently enabling the regeneration of collagen fibrils that mimic the architecture of the healthy corneal stroma (i.e., restoring the mechanical strength of the corneal tissues) 56.

It is known that the structural integrity of the endothelium is essential in maintaining corneal transparency and metabolic activity. Moreover, the endothelium is metabolically depended on fluid pump that responds to fluid leakage and also serves as a barrier to solutes from the anterior chamber 57. A high IOP, which affects the metabolic active-pumping mechanism, can therefore induce changes in the morphology and structure of the endothelium. Based on this hypothesis, scanning electron microscopy (SEM) was carried out to examine changes in the endothelium of glaucomatous eyes in response to the topical administration of Y-27632-loaded HMCNs with different shell thicknesses. As shown in Figure 5C, healthy endothelial cells (Pre group) were observed to have flat and hexagonal shapes, and interdigitating cellular borders were perceived, demonstrating the compact and robust structure of the normal rabbit endothelium. For rabbits induced with glaucoma, the continuous honeycomb structure of the endothelium was disrupted (GL group), and this developed to higher levels of severity in the progressively diseased eyes (Ctrl group), as verified by the presence of damaged and deformed endothelial cells and the loss of structural integrity. Based on the SEM images and IOP results, structural disruptions can therefore be ascribed to an elevated IOP, which exerts high stress on the endothelium, inducing cell damage and integration. Due to their limited drug loading and release capabilities, formulations using HMCNs with thicknesses of both 10 and 40 nm were not able to prevent the progressive damage of the endothelium, demonstrated by comparing the morphologies of the Y/CNP10 nm and Y/CNP40 nm groups to those of the Ctrl group. In contrast, corneal endothelial cells in the Y/CNP20 nm group revealed morphorlogy and structure similar to those in the healthy cornea (Pre group). This regenerative effect can be ascribed to sustained release of Y-27632, which could promote cell proliferation, enhance cell adhesion, and thereby facilitate the regeneration of corneal endothelial cells 58.

To support the SEM morphological observations, and knowing that the activity of Na^+^, K^+^-adenosine triphosphatase (ATPase) pumps situated in the basolateral membrane of corneal endothelial cells is strongly influenced by their structural integrity 59, quantitative real-time RT-PCR was performed to detect the gene expression patterns of ATP1A1 mRNA. Figure 5D indicates the ATP1A1 mRNA levels in the endothelium tissues of the test rabbit eyes. The obtained expression level in healthy (Pre) endothelium was set as 100%. Reduced expression levels were recorded in the diseased rabbit eyes, suggesting that the endothelial pump function was altered. The higher expression level in the Y/CNP20 nm group (in comparison to the Y/CNP10 nm and Y/CNP40 nm groups) further confirms the important role that HMCN carrier shell thickness has in repairing the corneal endothelium structure from elevated IOP-induced damage.

**Figure 6:**
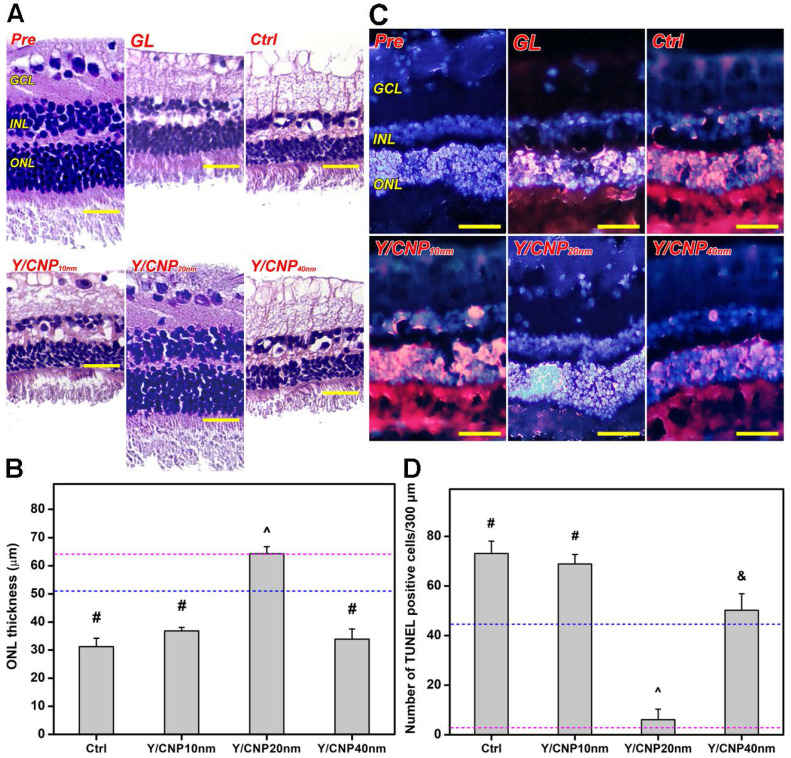
***In vivo* studies on shell thickness effects on retinal structure of glaucomatous eyes.** (A) Representative histological images of healthy (Pre), glaucomatous (GL), untreated (Ctrl), and treated (Y/CNP10 nm, Y/CNP20 nm, and Y/CNP40nm) glaucomatous retinas. Sections are stained with H&E. Scale bars: 50 µm. (B) Thickness of outer nuclear layer in different retinas; pink and blue dash lines showing the values of Pre and GL groups, respectively. Values are mean ± SD (n = 6). ^#^P < 0.05 vs Pre, GL, and Y/CNP20 nm groups; ^^^P < 0.05 vs GL, Ctrl, Y/CNP10 nm and Y/CNP40 nm groups. (C) Typical histological images of retinas from different rabbit eyes. Sections are stained with H&E and TUNEL. Red fluorescence is TUNEL positive nuclei staining. Scale bars: 80 µm. (D) Number of apoptotic TUNEL-positive cells/300 µm; pink and blue dotted lines showing the values of Pre and GL groups, respectively. Values are mean ± SD (n = 6). ^#^P < 0.05 vs Pre, GL, CNP20 nm, and CNP40 nm groups; ^^^P < 0.05 vs GL, Ctrl, CNP10 nm, and CNP40 nm groups; ^&^P < 0.05 vs Pre, Ctrl, CNP10 nm, and CNP20 nm groups. Ocular tissues were harvested from test rabbit eyes at 10 days post single administration (50 µL of Y-27632-loaded HMCN solution, a mixture of 2% w/v Y-27632 and 1 mg/mL of HMCNs).

As glaucoma progression involves retinal degeneration 60, the histology of the retinal sections of all test rabbit eyes was examined to further evaluate the relationship between the shell thickness of the HMCN carriers and pharmacological treatment efficacy. Figure 6A shows histological images of retinal sections from healthy (Pre), glaucomatous (GL), progressively glaucomatous (Ctrl), and treated glaucomatous (Y/CNP10 nm, Y/CNP20 nm, and Y/CNP40 nm) eyes. A typical retinal architecture, consisting of a ganglion cell layer (GCL), inner nuclear layer (INL), and outer nuclear layer (ONL), was observed in the normal retina section. In comparison to the Pre group, a substantial reduction in the thickness of GCL, INL and ONL in the GL and Ctrl groups was observed, confirming the alteration of retinal structure and characteristics in glaucomatous eyes 61. No impact to the diseased retina was observed in the Y/CNP10 nm and Y/CNP40nm groups, whereas the Y/CNP20 nm formulation was able to restore the retinal structure to a level close to that in the healthy eyes.

To support this observation, ONL thickness was quantified for all the groups (Figure 6B). Normal ONL thickness is ~64 µm, whereas the thickness of glaucomatous retinas is considerably reduced to ~51 µm. After ten days of topical administration, the ONL thicknesses of untreated and treated glaucomatous eyes were 31.2 ± 3.0 µm (Ctrl), 36.8 ± 1.3 µm (Y/CNP10 nm), 64.3 ± 2.5 µm (Y/CNP20 nm), and 33.9 ± 3.6 µm (Y/CNP40 nm), respectively. These results suggest the pharmacological treatment using Y-27632-loaded HMCNs with a shell thickness of 20 nm can effectively mitigate the progression of glaucoma and restore retinal structure, possibly due to the synergistic combination of the high performance in sustained and targeted delivery of Y-27632 and the strong potency of intrinsically anti-oxidant and anti-inflammatory HMCNs in the eye drop formulation. The level of photoreceptor cell apoptosis in the ONL of retinal sections was further evaluated using a TUNEL assay. Fluorescence images (Figure 6C) indicates the prominence of TUNEL-labeled (red) apoptotic cells in all groups. The prominence of red fluorescence signals (comparable to that in the GL and Ctrl groups) in the CNP10nm group demonstrates the ineffectiveness of the formulation using HMCNs with a thickness of 10 nm. The presence of red fluorescence signals was reduced in the Y/CNP40 nm group, and decreased significantly in the Y/CNP20 nm group, suggesting that the prevention of photoreceptor cell apoptosis led to the efficient mitigation of progressive glaucoma. Quantification of TUNEL positive cells in the test retinal sections was executed to support the qualitative evaluation that was based on the fluorescence images (Figure 6D). The values for apoptotic photoreceptor cells in the ONL of the healthy retina were as low as 2.8 ± 1.5 cells/300 µm, whereas values for the glaucomatous retina were 45.4 ± 8.9 cells/300 µm. The apoptotic cells in the progressively glaucomatous retina increased considerably to 73.1 ± 5.0 cells/300 µm, whereas decreased levels of apoptotic cells were detected in the treated retinas. The number of apoptotic photoreceptor cells in the Y/CNP20 nm group was significantly lower (6.1 ± 4.2 cells/300 µm), approaching the number in the healthy group. These data suggest the clear effects of HMCN carrier shell thickness on pharmacotherapy efficacy: the thinnest and thickest shell carriers could not effectively attenuate the progression of glaucoma, whereas the moderately thick shell carriers were able to rescue glaucoma in the rabbit model by restoring damaged photoreceptor cells in the retina. This regeneration effect can be ascribed to the improved bioavailability of both ROCK inhibitor Y-27632 drug and anti-oxidant/anti-inflammatory nanoceria in the intraocular tissues (Table 1) by the HMCN carriers with the moderate shell thickness. These data are further supported by previous studies that demonstrate photoreceptor cells in the retina can be protected and regenerated via inhibition of cell apoptosis, reduction of elevated ROS levels, and down-regulation of inflammatory molecules 14, 62-64.

Given that progressive glaucoma involves degeneration of optic nerve (i.e., loss and damage of retinal axons) and Y-27632 could improve axon regeneration 65, cross sections of optic nerves of test rabbit eyes were examined using transmission electron microscopy (TEM) to observe changes of axon morphology in response to the topical instillation (Figure S9A). The TEM image of the healthy optic nerve (Pre group) exhibits the normal morphology of retinal axons with high density and their myelin sheaths are uniform. A considerable change in retinal axons was found in the GL group, as signified by the swollen axons, low axon density, and thin myelin sheaths (i.e., glaucoma-induced degeneration of the optic nerve). The single dose instillation of various formulations behaves differently, indicating the pharmacological regulation of retinal axons in response to the shell thickness of the HMCN carriers. As compared to GL group, lesser axon density was presented in the Ctrl, Y/CNP10 nm, and Y/CNP40 nm groups. On the other hand, relatively condensed arrangement of the retinal axons with uniform myelin sheaths and greater axon density could be obtained using the Y/CNP20nm formulation. Quantified data for the axon density of the rabbit optic nerves were shown in Figure S9B. The axon density in healthy optic nerves was 8.3 ± 0.7 × 10^4^ axons/mm^2^. For glaucomatous optic nerves, the density was decreased to 5.3 ± 0.3 × 10^4^ axons/mm^2^ (GL group), suggesting the axon degeneration in glaucomatous eyes 66. Pharmacological treatments using Y/CNP10nm and Y/CNP40nm formulations could not prevent the decrease in axon density. On the other hand, the axon density in Y/CNP20nm group after 10 days of single dose instillation was comparable to that of healthy eyes (Pre group), implying the pharmacological effectiveness of the Y/CNP20 nm. This can be ascribed to the superior drug delivery performance of the anti-oxidant and anti-inflammatory HMCN carriers with shell thickness of ~20 nm that can retain high concentrations of Y27632 and nanoceria in the retina of the glaucomatous eyes, consequently facilitating the regeneration/repair of the damaged axons in the glaucomatous optic nerve. Considering that TM cells can be differentiated into retinal ganglion cells (RCGs) *in vitro* 67, investigation on the differentiation of TM cells into RGCs and their translocation to retina may be a promising approach for pharmacological assessment of a newly developed ophthalmic formulation-mediated retina and axon regeneration in future clinical translation.

## Conclusion

In summary, we have explored the effects of hollow ceria nanoparticle shell thickness on the targeted drug delivery of ROCK inhibitor Y-27632, and on the application of HMCNs as nano eye drops for the pharmacological treatment of glaucoma. The hollow nanoparticles with thicker shells possess greater abilities in inhibiting hydrogen peroxide-induced ROS generation, and in reducing high concentrations of IL-6 inflammatory molecules in the trabecular meshwork cells. The effects of different shell thicknesses are key considerations in regulating the drug release profile of the hollow nanoparticles: it has been demonstrated that a sustained release performance (over ten days) can be achieved for nanoparticles with a moderate shell-thickness of 20 nm. In a rabbit model of experimental glaucoma, the topical administration of nanoparticles loaded with Y-27632 demonstrated a strong correlation between shell-thickness and treatment efficacy. A single instillation of the Y-27632-loaded ceria nanoparticles with a shell thickness of 20 nm can achieve effective alleviation of the progression of glaucoma by simultaneously repairing the defective corneal stroma and corneal endothelium and restoring the photoreceptor cells in ONL from ocular hypertension-induced degeneration. Our findings, therefore, comprehensively demonstrate the importance of hollow ceria nanoparticle cell thickness in developing long-acting nano eye drops for the efficient management of glaucoma.

## Supplementary Material

Supplementary figures and tables.

## Figures and Tables

**Table 1:** The amount of Ce and Y-27632 in corneal, ciliary body, and retinal tissues of glaucomatous rabbit eyes treated with different drug-loaded HMCN formulations

Sample code	Ce			Y-26732		
	Cornea (μg/g)*^a^*	Ciliary body (μg/g)*^b^*	Retina (ng/g)*^c^*	Cornea (μg/g)*^d^*	Ciliary body (μg/g)*^e^*	Retina (ng/g)*^f^*
Y/CNP_10nm_	4.5 ± 0.8	50.3 ± 1.7	246.7± 19.3	0.4 ± 0.2	1.3 ± 0.5	28.3 ± 9.3
Y/CNP_20nm_	2.7 ± 1.1*^g^*	60.6 ± 2.4*^g^*	153.1± 22.8*^g^*	1.1 ± 0.2*^g^*	14.6 ± 1.9*^g^*	81.5 ± 11.2*^g^*
Y/CNP_40nm_	1.8 ± 0.9*^g^*	69.1 ± 3.0*^g^*	92.0 ± 27.0*^g^*	0.2 ± 0.1	1.8 ± 0.7	32.6 ± 7.4

*a:* Inductively coupled plasma mass spectrometry was employed to quantify the amount of ceria in the corneal tissues of test rabbit eyes. Data are expressed as mean ± SD (n = 6); *b:* Inductively coupled plasma mass spectrometry was employed to quantify the amount of ceria in the ciliary body tissues of test rabbit eyes. Data are expressed as mean ± SD (n = 6); *c:* Inductively coupled plasma mass spectrometry was employed to quantify the amount of ceria in the retinal tissues of test rabbit eyes. Data are expressed as mean ± SD (n = 6); *d:* High performance liquid chromatography was used to determine the amount of Y-27632 in the corneal tissues of test rabbit eyes. Data are expressed as mean ± SD (n = 6); *e:* High performance liquid chromatography was used to determine the amount of Y-27632 in the ciliary body tissues of test rabbit eyes. Data are expressed as mean ± SD (n = 6); *f:* High performance liquid chromatography was used to determine the amount of Y-27632 in the retinal tissues of test rabbit eyes. Data are expressed as mean ± SD (n = 6); *g:* Significant difference as compared to the CNP_10nm_ groups (P < 0.05).
