# Supplementary material for: Harnessing the tunable cavity of nanoceria for enhancing Y-27632-mediated alleviation of ocular hypertension
Source: Theranostics. 2021 Mar 13;11(11):5447–63. doi: 10.7150/thno.54525 (PMC8039939; doi:10.7150/thno.54525)
Supplement: Supplementary file 1 — Supplementary figures and tables. [file thnov11p5447s1.pdf]

# Supplementary Material

# Harnessing the tunable cavity of nanoceria for enhancing Y-27632-mediated alleviation of ocular hypertension

Li-Jyuan Luo[1*], Duc Dung Nguyen[1*], and Jui-Yang Lai[1,2,3,4]✉

1. Graduate Institute of Biomedical Engineering, Chang Gung University, Taoyuan 33302, Taiwan.
2. Department of Ophthalmology, Chang Gung Memorial Hospital, Linkou, Taoyuan 33305, Taiwan.
3. Department of Materials Engineering, Ming Chi University of Technology, New Taipei City 24301, Taiwan.
4. Research Center for Chinese Herbal Medicine, College of Human Ecology, Chang Gung University of Science and Technology, Taoyuan 33303, Taiwan.

*Both authors contributed equally to this work.

✉ Corresponding author: E-mail: jylai@mail.cgu.edu.tw.

## 1. Methods

### 1.1. Synthesis of surface modified and shell-thickness controlled HMCNs

Silica nanoparticles were first prepared by a sol-gel method and then used as templates for synthesis of the HMCNs. Briefly, TEOS (16 mL) was mixed with ethanol (560 mL) and the mixture was kept under stirring conditions. Subsequently, deionized water (112 mL) and ammonium hydroxide (16.8 mL) were added to the mixture. The resultant mixture in a flask container was sealed and kept at ambient conditions. After being stirred for one day, the mixture was consecutively centrifuged (10000 rpm, 10 min) and dispersed in water and ethanol, and finally heated at 65 °C for 6 h in an oven for obtaining silica nanotemplates [1].

For synthesis of shell-thickness controlled HMCNs, silica nanotemplates (600 mg) were dispersed in ethylene glycol (90 mL) in a ultrasonication bath. To this silica dispersion, 1 M cerium nitrate (4.5 mL) was added followed by stirring for 10 min. Subsequently, the mixture was placed in an autoclave, heated to 130 °C for 7 h. Once the solvothermal reaction terminated by cooling down to room temperature, the silica@ceria core-shell nanoparticles were collected by centrifugation (15000 rpm, 10 min) followed by washing with excess amount of ethanol. To obtain HMCNs with tunable shell thickness, a two-step process was employed. In the first step, the nanotemplate cores were removed by dispersing the silica@ceria nanoparticles in a sodium hydroxide solution (5 M) for 2 days at room temperature; during this removal process, the sodium hydroxide solution was substituted by the fresh one after 1 day. Subsequently, the mixture was consecutively centrifuged (10000 rpm, 10 min), dispersed in water and then in ethanol, and dried in air, consequently achieving hollow ceria nanoparticles as the starting material for the subsequent synthetic process. In the second step, the starting hollow nanoparticles, serving as hollow nanotemplates, were treated in the same solvothermal conditions as applied for the silica nanotemplates. By repeating the solvothermal deposition process, the HMCNs with tunable shell thickness could be achieved.

To generate targeted delivery feature for the shell-thickness controlled HMCNs, their surfaces were modified with chitosan and ZM241385. In short, HMCNs were first functionalized with phosphonate-PEG-COOH by mixing the two materials (HMCNs/phosphonate-PEG-COOH, 1/10 w/w) in deionized water and the mixed solution was adjusted to obtain pH of 3. Subsequently, excess molecules were removed via an ultrafiltration process (30 kDa, Merck Milipore). The phosphonate-PEG-COOH functionalized HMCNs were dispersed in deionized water. Next, the surface modification was executed using EDC chemistry that allowed covalent binding of chitosan and ZM241385 to the PEGylated HMCNs. Specifically, the PEGylated HMCNs (2 g) was dissolved in a MES buffer (10 mL) containing EDC (0.52 g) under agitation for 6 h, followed by mixing with chitosan (3 g) and ZM241385 (1.5 g) in MES buffer (10 mL). This reaction was proceeded at room temperature for 1 day and the resultant mixture was precipitated at 50 °C. The chitosan/ZM241385 modified HMCNs were finally lyophilized at -50 °C and stored in an enclosed vessel at ambient conditions.

### 1.2. Materials characterization

A transmission electron microscope (TEM, JSM-1200EX II JEOL) was employed to observe the nanostructures of surface modified and shell-thickness controlled HMCNs. A Bruker AXS D8 Advance X-ray diffractometer (Karksruhe, Germany) was used for crystallographic characterization; X-ray diffraction (XRD) patterns of the HMCNs were obtained over a 2θ scan range of 10 – 80° with a rate of 1°/min and a step size of 0.3° under Cu Kα radiation operated at 40 kV and 30 mA. DLS and zeta potential of the HMCNs were measured using Doppler microelectrophoresis (Zetasizer Nano ZS, Malvern Instruments, Worcestershire, UK). The specific surface area of HMCNs was measured with nitrogen adsorption/desorption (micromeritics, ASAP 2010) and calculated based on Brunauer-Emmett-Teller (BET) theory. To quantify the amount of free amino groups on unreacted chitosan residues, the ninhydrin assay was utilized. The test specimens of HMCNs were weighed and heated with a ninhydrin solution for 20 min. After cooling down and diluting the test solutions with 95% ethanol, their

optical absorbance at 570 nm was measured by a UV-Visible spectrophotometer (Thermo Scientific, Waltham, MA, USA) and standard absorbance curves of glycine. Considering that the amounts of free amino groups in the feeding chitosan and unreacted chitosan after the EDC chemistry are related to the optical absorbance of the solutions, their difference in absorbance was correlated to the amount of surface-bound amine groups on the HMCNs (n = 5). To determine ZM241385 amount, the surface modified and shell-thickness controlled HMCNs were dispersed in deionized water and centrifuged to collect the supernatant and ZM241385 was determined by UV absorbance measurement (n = 5).

### 1.3. In vitro biocompatibility studies

Human TM cell line was obtained from American Type Culture Collection (Manassas, VA, USA). The TM cell cultures were maintained in low glucose Dulbecco's Modified Eagle's Medium (DMEM) with L-glutamine and 110 mg/L sodium pyruvate supplemented with 10% fetal bovine serum (FBS), 100 μM non-essential amino acids, 100 units/mL penicillin, 100 μg/mL streptomycin sulfate, and 0.25 μg/mL amphotericin B at 37 °C in 5% $CO_2$ [2]. Only healthy TM cells from passages 5 through 8 were used for studies thereafter. The TM cells ($5 \times 10^4$ cells/well) were seeded into 24-well plates by 1 mL/well. Each well was divided into two compartments by cell culture inserts (Falcon 3095, Becton Dickinson Labware, Franklin Lakes, NJ, USA). The medium was then replaced with fresh culture medium containing each type of the HMCNs; in all in vitro experiments, the HMCN concentration in each cell culture was 150 μg/mL. Cell cultures grown in the absence of HMCNs served as control (Control) group for comparison. After incubation of the TM cultures with each type of the HMCNs for 2 days, cellular morphology was observed by a phase-contrast microscope (Nikon, Melville, NY, USA). Moreover, the metabolic activity was estimated by a mitochondrial dehydrogenase (MTS) assay. A microplate spectrometer was utilized to determine the absorbance at 490 nm. The results were expressed as relative MTS activity compared to that of the Control (n = 6). Comet assay was further used to assess the genotoxicity in

TM cells that exposed to the HMCNs. After lysing cells, the slides were subjected to optimized electrophoresis conditions and stained with DAPI for imaging using a fluorescence microscope (Axiovert 200 M, Carl Zeiss, Oberkochen, Germany). Quantitative analysis of the DNA damage for each cell was performed by the calculation as follow: comet tail length (μm) = maximum total length – head diameter. For obtaining the data, 200 cells were arbitrarily chosen and the comet tail lengths were averaged (n = 6).

### 1.4. Anti-oxidant and anti-inflammatory activity studies

For assessment of anti-oxidant activities of the surface modified and shell-thickness controlled HMCNs against oxidative stress, the TM cells were maintained in DMEM, 10% FBS, and antibiotic agents. Intracellular reactive oxygen species (ROS) was measured by staining the cells with 10 μM 2′,7′-dichlorodihydrofluorescein diacetate (DCFH-DA) (Molecular Probes, Eugene, OR, USA) at 37 °C for 1 h. Fluorescence micrographs (Ex. 488nm; Em. 525nm) was captured by a fluorescence microscope (Axiovert 200M; Carl Zeiss, Oberkochen, Germany). The fluorescence reading was recorded via a multimode microplate reader (BioTek Instruments, Winooski, VT, USA) to identify the fluorescence intensity differences (n = 4). Before exposing to hydrogen peroxide, the TM cells ($5 \times 10^4$ cells/well) were seeded in 24-well plates and treated with 150 μL of each type of the HMCN dispersions (1 mg/mL) for 1 day. The cell cultures were then further incubated in medium containing 200 μM hydrogen peroxide for 24 h. The cultures treated with 0 (Control group) or 200 (HP group) μM hydrogen peroxide for 1 day without 1-day pretreatment with any type of HMCNs were used for comparison [3]. In addition, free radical scavenging activity of HMCNs was also evaluated using the DPPH method [3]. Aqueous solutions were respectively obtained by dispersing 250 mg of each type of HMCNs in deionized water (12.5 mL), followed by adding an equal volume of an ethanol solution containing 1 mg of DPPH radical. After 30 min of incubation at 25 °C, the absorbance at 517 nm of the resulting dispersion was measured by the UV−Vis spectrophotometer. The DPPH scavenging activity (%) was

calculated to be $((A_0 - A_1)/A_0) \times 100$, where $A_0$ is the absorbance of blank DPPH solution that was used under the same reaction conditions in the absence of the HMCNs, while $A_1$ is the absorbance of DPPH solution in the presence of the HMCNs ($n = 5$).

Anti-inflammatory activities of different types of HMCNs (1 mg/mL) were determined by enzyme-linked immunosorbent assay (ELISA). The TM cells ($5 \times 10^4$ cells/well) were seeded in 24-well plates containing regular growth medium followed by an overnight incubation. For TGF-β stimulation, the medium was replaced with the fresh one containing 5 ng/mL. Each well of a 24-well plate was divided into two compartments by cell culture inserts (Becton Dickinson Labware). Different HMCN dispersions were individually added to the inner well at 37 °C to examine the TGF-β-stimulated cultures that were exposed to the test HMCNs (1 mg/mL). Unstimulated and TGF-β-stimulated TM cells without incubation with any substance respectively served as the negative control (NC) and positive control (PC) groups. After 3 days of incubation, IL-6 released from untreated/treated cells into the medium was detected by the Quantikine ELISA kit (R&D Systems, Minneapolis, MN, USA). Supernatants in each well were collected and cytokine bioassays were carried out according to instructions from the manufacturer [4]. Multiskan Spectrum Microplate Spectrophotometer (ThermoLabsystems) was used to record photometric readings at 450 nm ($n = 6$).

### 1.5. In vitro drug release profiles and cellular regulation properties

Drug loading content of different types of HMCN carriers was assessed by mixing the carrier dispersions (1 mg/mL) with Y-27632 (2% w/v) followed by adding the mixture into a vial containing 1.5 mL of BSS at pH value of 5.5. Next, the resultant mixture was sonicated for 2 h and then stirred for 24 h at room temperature. Subsequently, a NaOH solution was used to adjust the pH value of the mixture to 7.4 and then the mixture was further stirred for 12 h. The Y-27632-loaded HMCNs were collected by centrifugation and washed scrupulously with BSS at pH of 7.4 to remove Y-27632 molecules physically absorbed on exterior particle surfaces. The amount of free Y-27632 in the supernatant

was quantified by a high-performance liquid chromatography (HPLC) system equipped with a L-2400 UV detector (Hitachi, Tokyo, Japan) and a Mighty RP-18 column (4.6 × 250.0 mm; Kanto Chemical, Tokyo, Japan). Briefly, 0.1 mL of 1M NaOH and 2.0 ml of chloroform were added to 0.1 mL of the supernatant in a 12-mL glass-stoppered tube as described elsewhere [5]. The separation was carried out on a reversed-phase column (a Capcell pak C18 UG-120 S-5, 150×4.6 mm I.D., Shiseido, Tokyo, Japan) and heated to 40 °C. The mobile phase was methanol/sodium perchlorate (20 mM), which was controlled at pH value of 2.5 using perchloric acid. The samples were eluted at a flow rate of 1.0 mL/min and the UV detector was set at 270 nm.

To evaluate the shell thickness effects of the nanoparticles on Y-27632 to modulate TM contraction in vitro, different Y-27632-loaded HMCN carriers were incubated with TM cells. The cells were serum-starved overnight and then stimulated with TGF-β (5 ng/mL), washed with PBS, fixed in 4% paraformaldehyde, and washed with PBS again. Subsequently, the TM cells were permeabilized for 10 min with 0.1% Triton X-100 and washed with PBS three times [6]. The cells were finally fluorescence-stained with DAPI, FITC-HMCNs, and phalloidin. The mean fluorescence intensities of the stains were analyzed using ImageJ software (n = 6). Shell thickness effects on the dephosphorylation of myosin light chain 2 (MLC2) and restoration of cofilin activity were examined using Western blots. After serum starving for 1 day, the TM cells were first pretreated with each of different Y-27632-loaded HMCNs for 30 min, and then stimulated with TGF-β for 24 h. Subsequently, the cells were washed three times with cold PBS, lysed with RIPA buffer (Thermo Scientific, Rockford, IL) containing protease (Thermo Scientific) and phosphatase inhibitors (Nacalai Tesque INC., Kyoto, Japan). Supernatant from cell extracts after centrifugation was collected and the protein content was determined using a BCA protein assay kit (Thermo Scientific). The specimens were resolved using SDS-PAGE and then transferred onto polyvinylidene difluoride membranes by electroblotting [7]. Next, the membranes were blocked with 2% ECL Advance Blocking Reagent (GE Healthcare, Little Chalfont, UK) in Tris-buffered saline

containing 0.1% Tween-20 (TBS-T) for 30 min, following incubation with primary antibodies diluted with 5% Bovine Serum Albumin (WAKO) in TBS-T overnight at 4°C. Finally, the membranes were incubated with horseradish peroxidase-conjugated anti-rabbit IgG (1:2,000 dilution; Cell Signaling Technology) or horseradish peroxidase-conjugated anti-mouse IgG (1:5,000 dilution; GE Healthcare), for 1 h at room temperature. Densitometry of immunoreactive bands was performed using the Image J software.

### 1.6. Shell thickness effects on glaucoma therapy

For comparison, glaucoma induction using latex microbeads was also employed. Specifically, the microbeads (a diameter of 15 µm; FluoSpheres; Invitrogen, Carlsbad, CA, USA) were first reformulated in phosphate buffered saline at a concentration of $5 \times 10^6$ beads/mL. Then 100 µL of the microbead dispersion was intracamerally injected (once per week) to the anesthetized rabbits (via the same anesthesia procedure for those induced with α- chymotrypsin) for a follow-up period of 4 weeks to achieve sustained and elevated IOP. Immediately after each microbeads injection, a discharge of 100 µL of aqueous humor was performed to avoid acute IOP spike. After injection, 0.3% ofloxacin ophthalmic solution (Oflovid; Santen Pharmaceutical Co., Osaka, Japan) was applied on the eye to prevent infection. IOP values were recorded in predetermined time intervals utilizing a Schiotz tonometer (AMANN Ophthalmic Instruments, Liptingen, Germany) ($n = 6$). IOP values for the normal eyes (in the range of 21.2 – 24.6 mmHg) were referred to as the baseline (zero-value) and presented IOP data (test/treated eyes) were specified as the change to baseline at each time point. For electroretinogram (ERG) measurements, the animals were anesthetized to avoid electrical noise from muscle activity [8]. Before the test, 1% cyclopentolate hydrochloride ophthalmic solution (Alcon) was topically applied on rabbit eyes to maximize their pupil dilation. After 30 min of dark adaptation, an Ag/AgCl electrode (Biopac Systems, Santa Barbara, CA, USA) was applied on the topically anesthetized cornea, a reference electrode on the ear, and a subcutaneous ground electrode on the neck of the animals. The ERG signals were

measured from each eye, and 10 responses to white light flashes (4 ms, 1 Hz) using a photic stimulator at maximum brightness (90 cd s/$m^2$ without a filter) were amplified and filtered. Electrophysiological responses were averaged for each run, and the mean of the five independent runs was used for subsequent analysis of the peak amplitudes of the a- and b-waves of the ERG (n = 6). For the final experiments (i.e., after 10 days of topical instillation), the animals were euthanized with $CO_2$ gas for harvesting ocular tissues. High performance liquid chromatography and inductively coupled plasma mass spectrometry were respectively employed to quantify concentration of Y-27632 and ceria resided in the corneal, ciliary body, and retinal tissues of test rabbit eyes (n = 6). For electron microscopy observations, corneal tissues were fixed by immersion in 2.5% glutaraldehyde in 0.1 M sodium cacodylate buffer, followed by aqueous 1% osmium tetroxide. For scanning electron microscopy (SEM) observation, the corneal endothelium was exposed to electron beam in a vacuum chamber of a SEM microscope. For transmission electron microscopy (TEM) observation, corneal stroma specimens were dehydrated in ethanol and embedded in Araldite CY212 resin. Sections of corneal stromal tissue specimens were then obtained by cutting the resin and located on copper grids and stained with uranyl acetate [9]. Young modulus of the cornea tissues (5 μm thick sections) was obtained using a universal testing machine (Instron, Canton, MA, USA) operating at a crosshead speed of 0.5 mm/min under ambient conditions (25 °C, humidity of 50%) until the tissue ruptured (n = 6). In addition, the rabbit corneal endothelium was further processed for quantitative real-time reverse transcription polymerase chain reaction (RT-PCR) analyses of ATP1A1 mRNA levels [10]. In other experiments, tissue samples were fixed in 4% paraformaldehyde. After removing anterior segments, the posterior eyecups were mounted onto cold chucks in embedding medium (OCT Tissue-Tek;Sakura Finetek, Torrance, CA, USA) and frozen at -70 °C. Frozen specimens were prepared with the use of a cryostat into 5 μm sections at -20 °C. Cryosections were cut along the vertical meridian of the eye through the optic nerve [8]. The thin sections were further stained with H&E and observed under a calibrated optical microscope (Nikon, Melville, NY, USA) to determine the thickness of the nuclear

layer (n = 6). Apoptotic photoreceptor cell number in retinal section was analyzed by terminal deoxynucleotidyl transferase (TdT)-mediated dUTP nick end labeling (TUNEL) assay (Roche Diagnostics, Indianapolis, IN, USA). After being fixed and permeabilized, the tissue specimens were treated with a mixed solution of TdT and fluorescein isothiocyanate dUTP in a humid chamber for 1 h at 37 °C. Slides were counterstained with 4′,6-diamidino-2-phenylindole (DAPI; Vector, Peterborough, England, UK) for identification of cell nuclei. Under a fluorescence microscope (Carl Zeiss), the number of TUNEL-positive apoptotic cell nuclei was counted at three randomly selected fields.

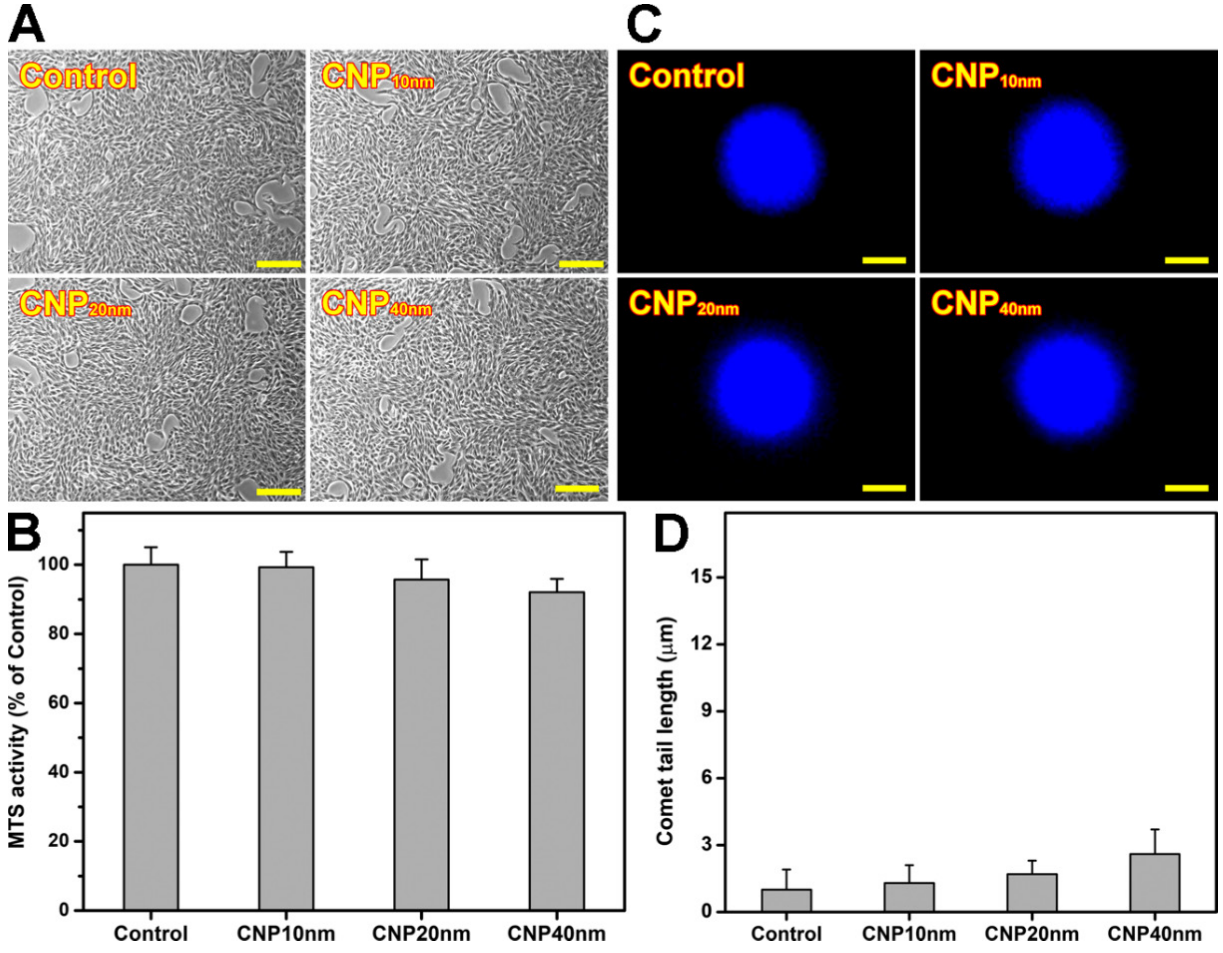


**Figure S1.** In vitro biocompatibility studies. (A) Phase-contrast micrographs and (B) MTS activity of TM cultures after a 2-day exposure to various HMCN carriers. Control: without materials; scale bars: 100 μm; results in (B) are expressed as percentage of Control; data are mean ± SD (n = 6). (C) Fluorescence photomicrographs and (D) tail lengths of comet assay of TM cultures after 2 days exposing to different HMCN carriers. Scale bars: 10 μm; data in (D) are mean ± SD (n = 6).

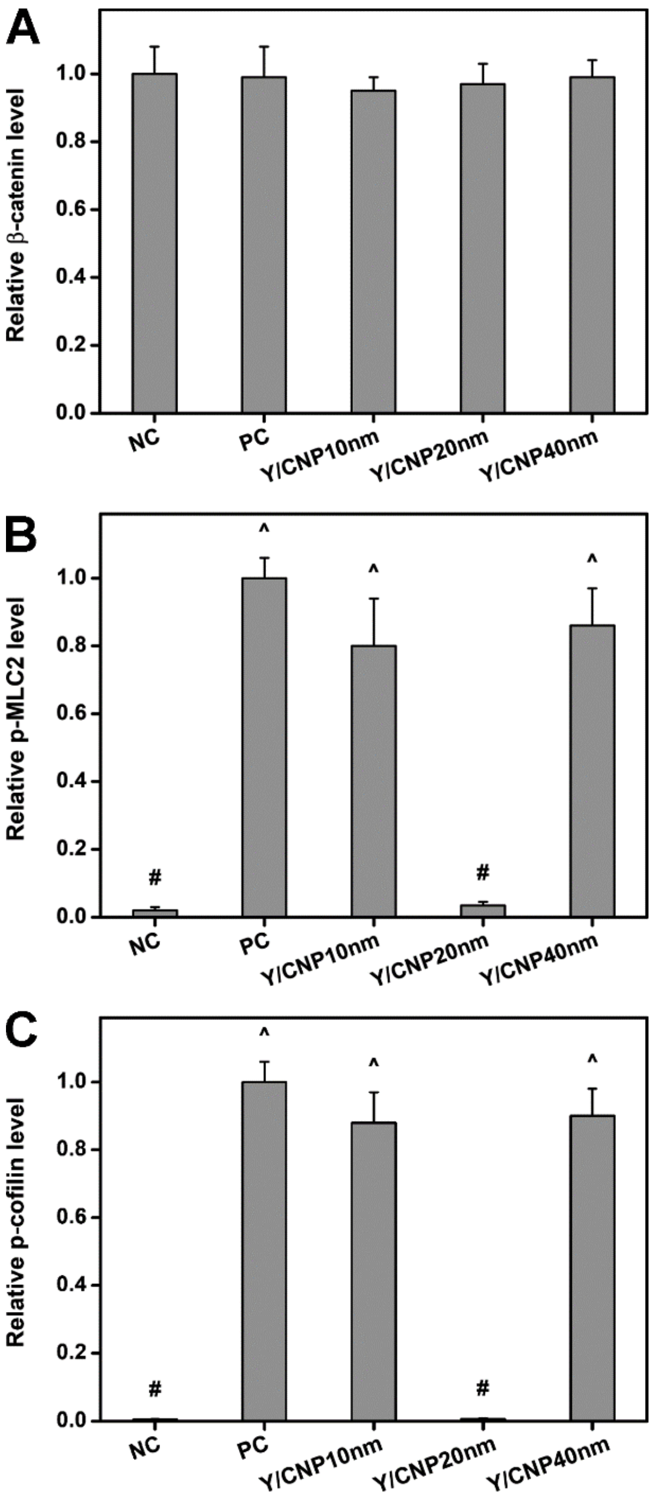


**Figure S2.** Relative (A) β-catenin, (B) p-MLC2, and (C) p-cofilin levels in the healthy (NC) and TGF-β-stimulated TM cells untreated (PC)/treated (Y/CNP10nm, Y/CNP20nm, and Y/CNP40nm) with Y-27632-loaded HMCNs with different shell-thicknesses. Values are mean ± SD (n = 6). #P < 0.05 vs PC, Y/CNP10nm, and Y/CNP40nm groups; ^P < 0.05 vs NC and Y/CNP20nm groups.

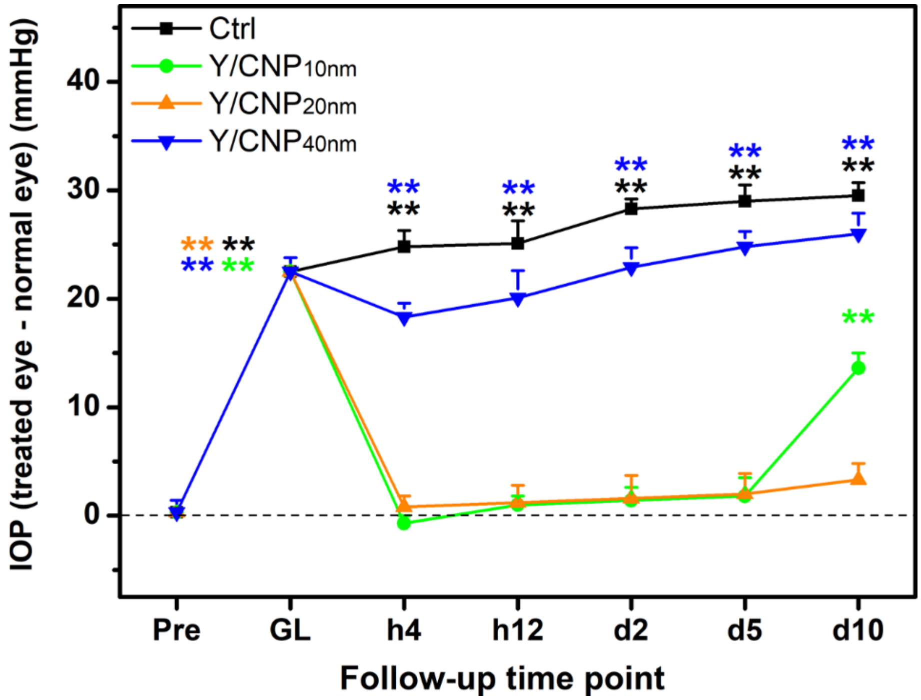


**Figure S3.** IOP measurements after topical administration of different types of Y-27632-loaded HMCNs into glaucomatous eyes (GL). Glaucomatous animals receiving no carrier and no drug serve as control groups (Ctrl). Asterisks indicate statistically significant differences. ($^{**}P < 0.005$; n = 6) as compared with the baseline IOP values. Follow-up time point: preoperation (Pre); hour (h); day (d).

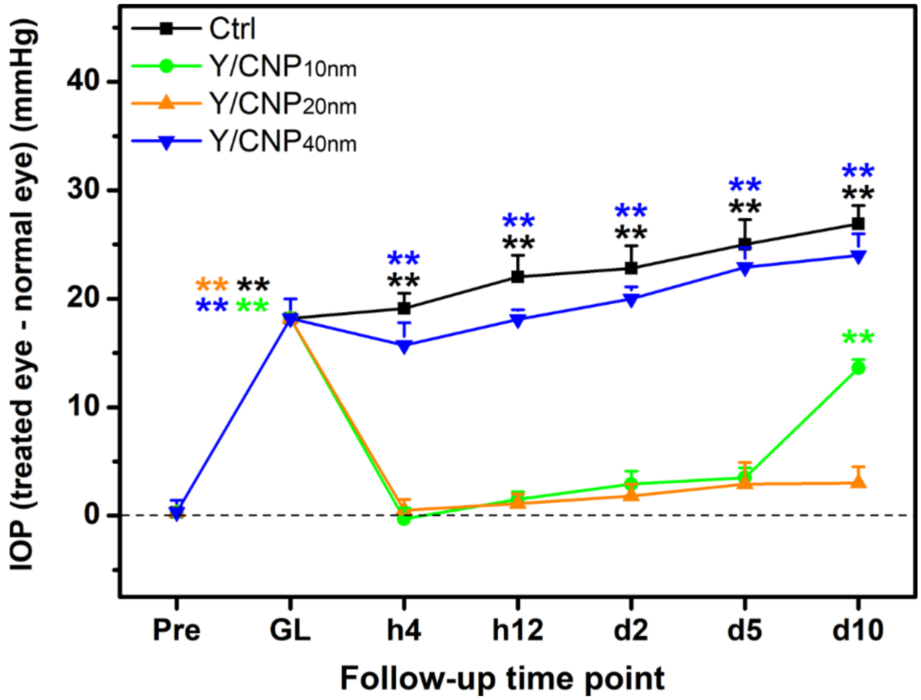


**Figure S4**. IOP measurements after topical administration of different Y-27632-loaded HMCN formulations into glaucomatous eyes (GL) induced by injection of latex microspheres. Glaucomatous animals receiving no carrier and no drug serve as control groups (Ctrl). Asterisks indicate statistically significant differences. (**$P < 0.005$; $n = 6$) as compared with the baseline IOP values. Follow-up time point: preoperation (Pre); hour (h); day (d).

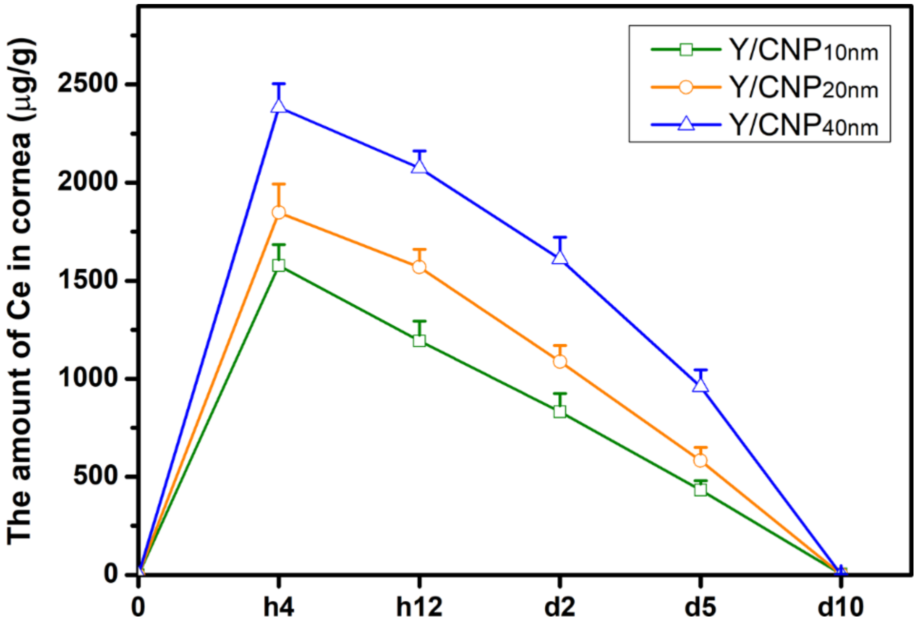


**Figure S5.** Time course of ceria concentrations in the cornea of test rabbit eyes after the single topical administration of different HMCN formulations (Y/CNP10nm, Y/CNP20nm, and Y/CNP40nm), respectively. Values are mean ± SD (n = 6). Follow-up time point: hour (h) and day (d).

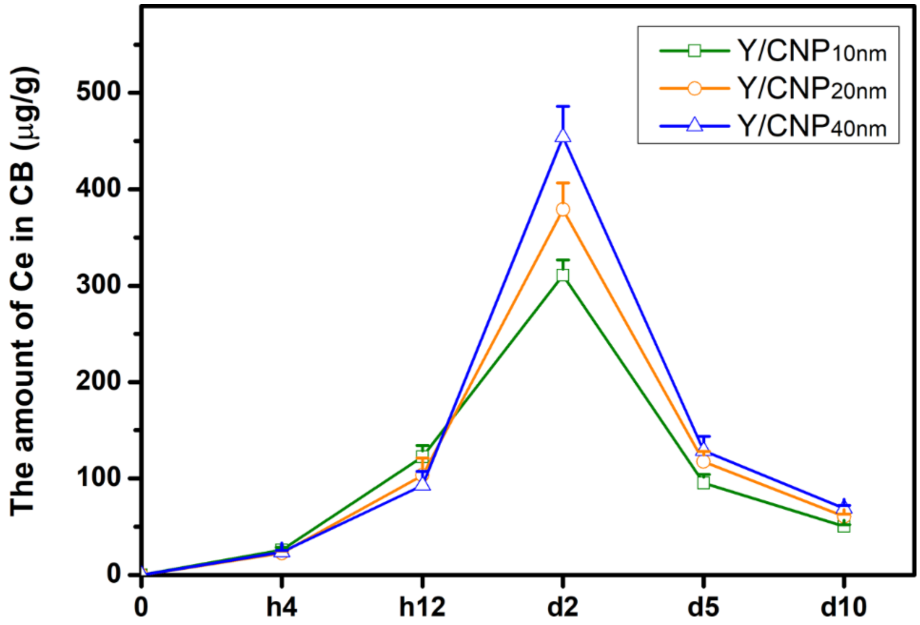


**Figure S6.** Time course of ceria concentrations in the ciliary body (CB) of test rabbit eyes after the single topical administration of different HMCN formulations (Y/CNP10nm, Y/CNP20nm, and Y/CNP40nm), respectively. Values are mean ± SD (n = 6). Follow-up time point: hour (h) and day (d).

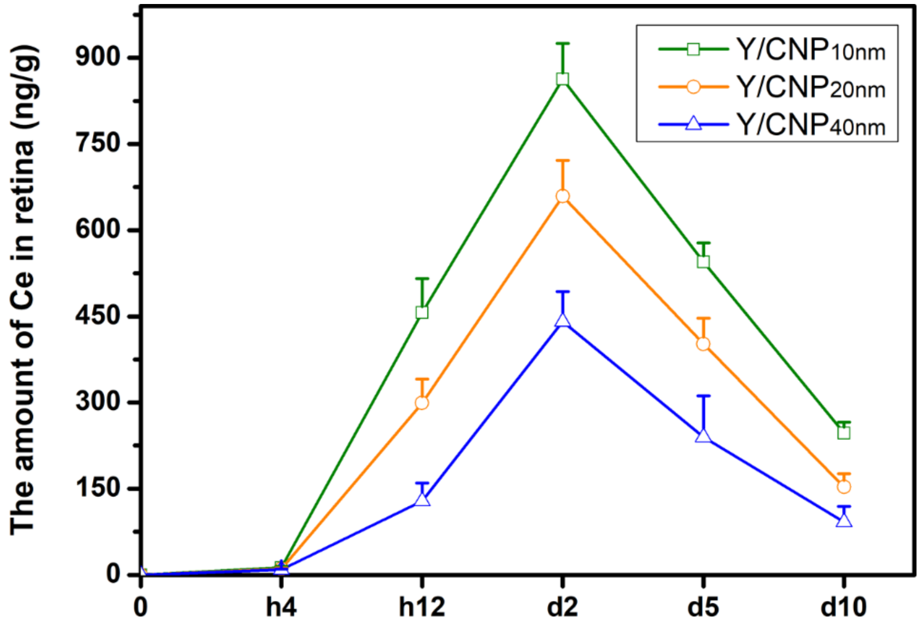


**Figure S7.** Time course of ceria concentrations in retina of test rabbit eyes after the single topical administration of different HMCN formulations (Y/CNP10nm, Y/CNP20nm, and Y/CNP40nm), respectively. Values are mean ± SD (n = 6). Follow-up time point: hour (h) and day (d).

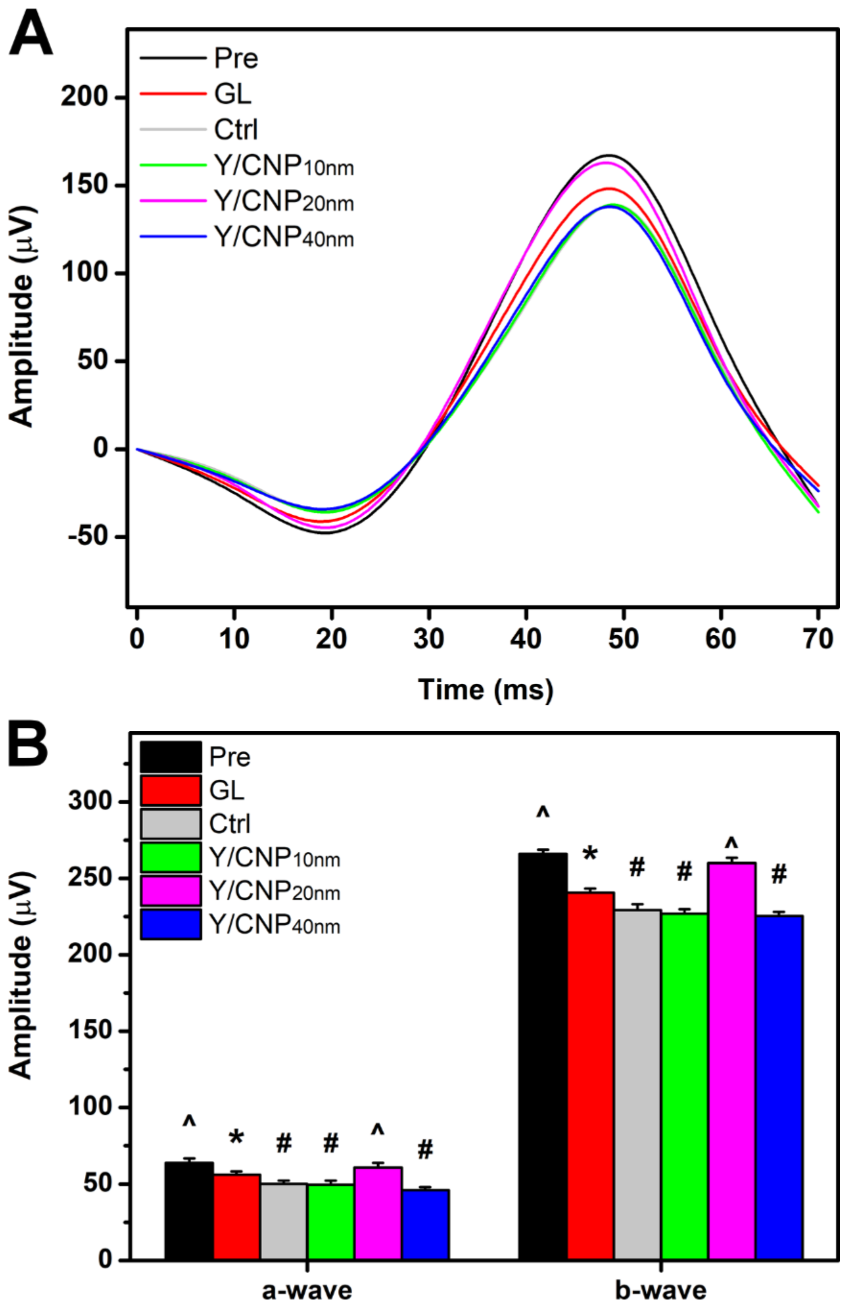


**Figure S8.** In vivo studies on the shell thickness effects. (A) Representative ERG recordings of rabbit eyes at preoperation (Pre) and those with experimentally induced glaucomatous optic neuropathy (GL) after different pharmacological treatments. Glaucomatous animals receiving no materials and drugs serve as control groups (Ctrl). (B) Quantification of a- and b-wave amplitudes. Values are mean ± SD (n = 6). $^{*}P < 0.05$ vs all group; $^{\#}P < 0.05$ vs Pre, GL, and Y/CNP20nm group; $^{\wedge}P < 0.05$ vs GL, Ctrl, Y/CNP10nm, and Y/CNP40nm groups.

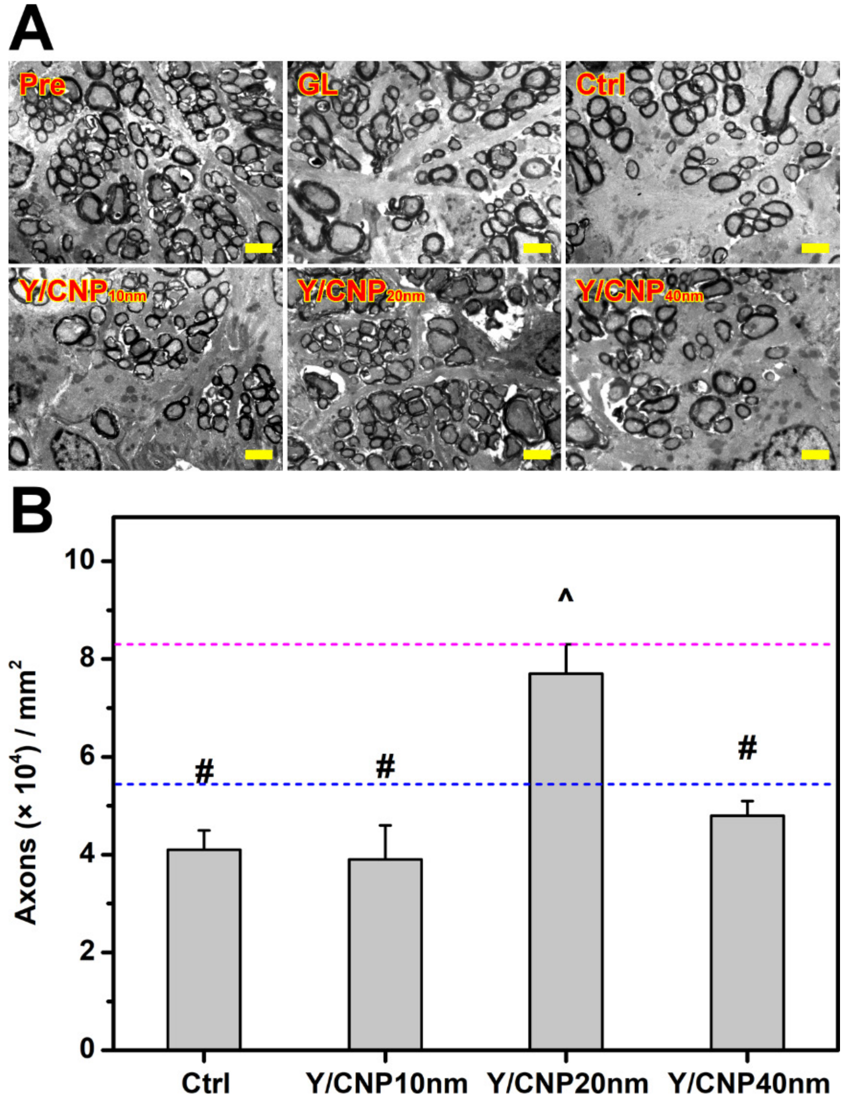


**Figure S9.** In vivo studies on the shell thickness effects of glaucomatous optic nerves. (A) Typical TEM images of optic nerves from rabbit eyes at preoperation (Pre group) and those afflicted with glaucoma before (GL group) and after the pharmacological treatment (Y/CNP10nm, Y/CNP20nm, and Y/CNP40nm groups). Glaucomatous animals receiving no materials and drugs served as the control groups (Ctrl). Scale bars: 1 μm. (B) Quantification of the axon density. Pink and blue dashed lines represent the axon densities of Pre and GL groups, respectively. The values are expressed as the mean ± SD (n = 6). $^{\wedge}P < 0.05$ vs GL, Ctrl, Y/CNP10nm, and Y/CNP40nm groups; $^{\#}P < 0.05$ vs Pre, GL, and Y/CNP20nm groups.

**Table S1.** DPPH free radical scavenging activity and material characterization of surface modified HMCNs

| Sample code | Inhibition of DPPH radical (%)[a] | Particle size (nm)[b] | Surface-bound amine group (μmoL/mg NP)[c] | ZM grafting amount (μg/mg NP)[d] |
|---|---|---|---|---|
| $CNP_{10nm}$ | 35.7 ± 2.8 | 70.3 ± 8.9 | 207.6 ± 9.3 | 6.9 ± 1.2 |
| $CNP_{20nm}$ | 42.7 ± 3.1[e] | 75.6 ± 8.4 | 225.7 ± 7.4[e] | 11.2 ± 1.5[e] |
| $CNP_{40nm}$ | 57.8 ± 2.9[e] | 83.1 ± 7.1 | 249.8 ± 9.8[e] | 16.4 ± 0.9[e] |

[a] Determination of free radical scavenging activity by 2,2'-diphenyl-1-picrylhydrazyl (DPPH) method. Data are expressed as mean ± SD (n = 5).

[b] Determination by Doppler microelectrophoresis. Data are expressed as mean ± SD (n = 5).

[c] Determination by ninhydrin assay. Data are expressed as mean ± SD (n = 5).

[d] Determination by UV absorbance measurement. Data are expressed as mean ± SD (n = 5).

[e] Significant difference as compared to the CNP10nm groups (P < 0.05).

## References


1. Strandwitz NC, Stucky GD. Hollow microporous cerium oxide spheres templated by colloidal silica. Chem Mater. 2009; 21: 4577-4582.
2. Liton PB, Li G, Luna C, Gonzalez P, Epstein DL. Cross-talk between TGF-beta1 and IL-6 in human trabecular meshwork cells. Mol Vis. 2009; 15: 326-334.
3. Lai JY, Luo LJ. Antioxidant gallic acid-functionalized biodegradable in situ gelling copolymers for cytoprotective antiglaucoma drug delivery systems. Biomacromolecules. 2015; 16: 2950-2963.
4. Lai JY, Lue SJ, Cheng HY, Ma DHK. Effect of matrix nanostructure on the functionality of carbodiimide cross-linked amniotic membranes as limbal epithelial cell scaffolds. J Biomed Nanotechnol. 2013; 9: 2048-2062.
5. Iizuka K, Shimizu Y, Tsukagoshi H, Yoshii A, Harada T, Dobashi K, et al. Evaluation of Y-27632, a Rho-kinase inhibitor, as a bronchodilator in guinea pigs. Eur. J. Pharmacol. 2000; 406: 273-279.
6. Yu N, Zhang Z, Chen P, Zhong Y, Cai X, Hu H, et al. Tetramethylpyrazine (TMP), an active ingredient of Chinese herb medicine chuanxiong, attenuates the degeneration of trabecular meshwork through SDF-1/CXCR4 axis. PLoS One. 2015; 10: e0133055.
7. Inoue-Mochita M, Inoue T, Fujimoto T, Kameda T, Awai-Kasaoka N, Ohtsu N, et al. p38 MAP kinase inhibitor suppresses transforming growth factor-β2-induced type 1 collagen production in trabecular meshwork cells. PLoS One. 2015; 10: e0120774.
8. Chou SF, Luo L J, Lai JY. In vivo pharmacological evaluations of pilocarpine-loaded antioxidant-functionalized biodegradable thermogels in glaucomatous rabbits. Sci Rep. 2017; 7: 42344.
9. Young RD, Swamynathan SK, Boote C, Mann M, Quantock AJ, Piatigorsky J, et al. Stromal edema in klf4 conditional null mouse cornea is associated with altered collagen fibril organization and reduced proteoglycans. Invest Ophthalmol Vis Sci. 2009; 50: 4155-4161.
10. Lai JY. Effect of chemical composition on corneal tissue response to photopolymerized materials comprising 2-hydroxyethyl methacrylate and acrylic acid. Mater Sci Eng C. 2014; 34: 334-340.
